# Cultivation of Asexual Intraerythrocytic Stages of *Plasmodium falciparum*

**DOI:** 10.3390/pathogens12070900

**Published:** 2023-07-01

**Authors:** Leonardo K. Basco

**Affiliations:** 1 Aix-Marseille Université, Institut de Recherche pour le Développement (IRD), Assistance Publique—Hôpitaux de Marseille (AP-HM), Service de Santé des Armées (SSA), Unité Mixte de Recherche (UMR) Vecteurs—Infections Tropicales et Méditerranéennes (VITROME), 13005 Marseille, France; lkbasco@yahoo.fr or leonardo.basco@ird.fr; 2 Institut Hospitalo-Universitaire—Méditerranée Infection, 19-21 Boulevard Jean Moulin, 13005 Marseille, France

**Keywords:** antimalarial drug resistance, in vitro assay, in vitro cultivation, malaria, *Plasmodium falciparum*

## Abstract

Successfully developed in 1976, the continuous in vitro culture of *Plasmodium falciparum* has many applications in the field of malaria research. It has become an important experimental model that directly uses a human pathogen responsible for a high prevalence of morbidity and mortality in many parts of the world and is a major source of biological material for immunological, biochemical, molecular, and pharmacological studies. Until present, the basic techniques described by Trager and Jensen and Haynes et al. remain unchanged in many malaria research laboratories. Nonetheless, different factors, including culture media, buffers, serum substitutes and supplements, sources of erythrocytes, and conditions of incubation (especially oxygen concentration), have been modified by different investigators to adapt the original technique in their laboratories or enhance the in vitro growth of the parasites. The possible effects and benefits of these modifications for the continuous cultivation of asexual intraerythrocytic stages of *P. falciparum*, as well as future challenges in developing a serum-free cultivation system and axenic cultures, are discussed.

## 1. Introduction

*Plasmodium* spp. constitute a wide variety of malaria parasites with different vertebrate hosts (reptiles, birds, rodents, non-human primates, humans) and insect vectors [1,2]. They are single-celled organisms in the Apicomplexa phylum, which includes many other obligate intracellular parasitic protozoans of medical and veterinary importance, such as coccidia and *Toxoplasma gondii*. As eukaryotes, malaria parasites, unlike prokaryotic bacteria, have membrane-bound intracellular compartments and no discrete cell wall outside their delicate limiting plasmalemma. *Plasmodium* spp. have a complex metabolism and, being parasites, depend for survival on the cells of the multicellular eukaryotic host (erythrocytes, liver parenchymal, or reticuloendothelial cells in the vertebrate stages or gut and salivary gland cells in the arthropod vector) and the host environment, which supply the basic components for biosynthesis [3].

Malaria parasites undergo several different cell cycles: (i) mitosis and meiosis (i.e., sexual reproduction) during sporogony in the mosquito vector, (ii) asexual hepatic and erythrocytic schizogony in the human host, and (iii) sexual differentiation into gametocytes in the human host [3,4,5]. After inoculation of the sporozoites by the mosquito vector, sporozoites enter the liver cells. Hepatic schizogony occurs without any clinical symptoms. The merozoites released from ruptured hepatic schizonts initiate intraerythrocytic schizogony, which may be associated with various clinical signs and symptoms. The division cycle in the blood (blood schizogony) is entirely within the erythrocyte. The erythrocytic schizogony goes through different developmental stages during the 48 h period (72 h period for *Plasmodium malariae*) in the human host [6,7]. The rings and trophozoites are the pre-nuclear division stages and, by definition, contain only one nucleus (under light microscopy, the presence of two nuclei is an artifact of a bilobed nucleus). Rings are flat or concave with a thin rim of organelle-rich cytoplasm encircling a large, flat, and clear center with few organelles. Trophozoites are much larger in size and have a thick cytoplasm and digestive vacuole containing hemozoin. Schizonts are defined morphologically as the developmental stage with >2 nuclei. In the literature, some authors distinguish between “pre-schizonts” and “schizonts” [8]. The term “pre-schizont” refers to schizonts with 3–8 nuclei. Mature schizonts refer to the more advanced stage (>8 nuclei), in which the parasite (especially *Plasmodium vivax*, *Plasmodium ovale*, and *P. malariae*) may occupy most of the intraerythrocytic space and the merozoites are segmented and individualized. When the infective merozoites escape from the erythrocyte at the end of this period, they are free in the plasma for only a brief period (probably seconds) [9]. The merozoite has special organelles (rhoptries, nicronemes, and dense granules) that facilitate its entry into the new host cell. Gametocytogenesis is initiated during the erythrocytic phase in the human host. The maturation of *Plasmodium falciparum* gametocytes takes about 10–12 days and occurs in the deep organs, notably in the bone marrow [10]. Mature gametocytes circulate in the peripheral circulatory system and are taken up by female *Anopheles* mosquitoes during subsequent blood meals. Sexual reproduction occurs in the mosquito gut.

As briefly described above, *Plasmodium* spp. have a complex metabolism. These facts are not ignored in the cultivation of malaria parasites in vitro. Here, the optimal host environment is artificially reproduced with greater or lesser success, depending on the species, in a laboratory setting under controlled conditions. Our knowledge of malaria biology and biochemistry is still incomplete, and precise nutritional requirements are not known, not even for the most successfully cultivated species, *P. falciparum*. The cultivation of *Plasmodium* spp. resembles most in its requirements the cultivation of viruses, which require complex host cells, rather than the cultivation of pathogenic bacteria, most of which will thrive on cell-free food sources such as serum, yeast extract, and meat broth. The development of short-term and long-term cultivation techniques for a blood-stage malaria parasite was more difficult to achieve than for many viruses, bacteria, and eukaryotic cells. Even now, of the four *Plasmodium* species found in humans, blood stages of *P. vivax*, *P. ovale*, and *P. malariae* have still not been continuously cultivated in vitro with any success (note: short-term cultures of *P. vivax* blood forms have been achieved and are proving to be informative on the distribution of drug resistance in blood stages of this species [11]; malaria due to *Plasmodium knowlesi*, the fifth malaria species that may infect humans, is a zoonosis [12]). It is probably best to concentrate on the human *Plasmodium* species with which we have had the most success, *P. falciparum*, when discussing particular features of in vitro cultivation. From the point of view of the most important forms of drug resistance, only the blood stages are of immediate interest, and these are the only stages where facile and routine cultures are achievable in most facilities.

The continuous culture of *P. falciparum* has had a major impact on almost all areas of malaria research [13]. It has become a reliable source of biological material for experiments in any properly equipped laboratory outside endemic regions and a means to obtain various fractions (DNA, RNA, proteins, and organelles) for basic science. More than 45 years after the development of in vitro techniques for long-term malaria parasite propagation, the basic technical procedures remain unchanged. However, a number of factors involved in the cultivation method can be and have been modified in an effort to enhance the efficacy of in vitro propagation rate. Over the years, these modifications have produced variations in recommended protocols in different specialized laboratories in the world.

The objective of the present work is to review the basic principles of the cultivation of the asexual intraerythrocytic stages of *P. falciparum*. This work is a traditional narrative literature review. The initial search for published papers was based on the search term “in vitro culture or cultivation *Plasmodium*” in the PubMed database with the aim to obtain the maximum number of relevant published articles. Other sources included books on malaria, book chapters, and references listed in selected papers. Papers written in English, French, Spanish, Portuguese, or Japanese were included. Except for the first published papers on in vitro cultivation of malaria parasites in 1911–1912, the search period spanned from January 1970 to February 2023. Most of the papers dealing with *Plasmodium* spp. other than *P. falciparum* were excluded unless they referred to the in vitro cultivation method developed by Trager and Jensen to illustrate the impact of this technique beyond *P. falciparum* [13]. Most early studies on cultivation before 1976 have been excluded. Readers interested in the “early history” of the development of cultivation methods of malaria parasites are referred to earlier reviews [14,15,16]. The cultivation of gametocytes, hepatic stages, and sporogonic stages is beyond the scope of this review.

## 2. Historical Background

Initial attempts to cultivate malaria parasites in vitro date back to the end of the 19th century, soon after the discovery of malaria parasites (*P. malariae*) in human erythrocytes by Charles Louis Alphonse Laveran (1845–1922), a French army surgeon, in Constantine, Algeria, in 1880. During the period between 1880 and 1911, only short-term survival, but not multiplication, was attained in vitro. The first successful attempts to grow the intraerythrocytic asexual stages of malaria parasites (*P. falciparum*, *P. malariae*, and *Plasmodium vivax*) were reported in 1912 [17,18]. The simple method developed by Bass and Johns consisted of the collection of venous blood from malaria-infected patients, defibrination, and the addition of d-glucose. The defibrinated whole blood is incubated at 40 °C and allowed to settle and separate into cellular components and plasma. Viable parasites undergo schizogony in the thin layer at the top of the red cell pellet for up to three complete generations (usually only for a single cycle, rarely four cycles) if leukocytes are removed. This method was adopted by investigators worldwide and became the reference for the short-term cultivation of human malaria parasites during the first half of the 20th century. The in vitro drug susceptibility test derived from the technique, called the “macrotest” or “macrotechnique”, was used until the 1980s [19,20]. However, some investigators were not able to reproduce Bass and John’s experiments and questioned whether the technique allows reinvasion and development of new generations of malaria parasites [14].

The report by Bass and Johns was shortly followed by World War I (1914–1917). Heavy casualties due to malaria among the troops deployed in southern Europe and a shortage of quinine, the only effective antimalarial drug available at that time, maintained the interest of governments and pharmaceutical companies in malaria research, notably for screening and development of new synthetic antimalarial compounds. During the 1920s and 1930s, German scientists at the Bayer–Meister–Lucius Laboratories synthesized and developed quinoline-based synthetic drugs, including pamaquine (also known as plasmoquine), mepacrine (also known as quinacrine or atebrin), sontoquine, and chloroquine. The antimalarial activity was screened by the in vivo avian malaria model. During the same period, many attempts were made by investigators to propagate the parasite blood stages in vitro, mostly avian, simian, and human malaria parasites, with different culture media and under various experimental conditions. However, the trial-and-error approach did not lead to successful continuous in vitro cultivation.

During World War II (1939–1945), malaria was recognized as a potentially serious medical problem with major repercussions on military operations both in the regions surrounding the Mediterranean Sea (i.e., southern Europe and North Africa, where malaria was endemic during that time) and Pacific Ocean. Quinine was again in shortage, partly due to the Axis occupation of The Netherlands and the Dutch colony of Java, the major producer of quinine at that time (note: quinine is readily obtained from *Cinchona* spp. tree bark; total chemical synthesis with the correct spatial configuration, i.e., stereoisomer, was achieved only at the turn of the twenty-first century but is uneconomical for mass production [21]). The synthetic drugs available at the outbreak of the war (i.e., mepacrine) were not well-tolerated by humans. Antimalarial drug screening and development programs were actively being pursued in Germany and France and were initiated in the United States of America, the United Kingdom, and Australia. These war-related research efforts led to the synthesis and development of chloroquine (synthesized in 1934 but developed later for clinical use), proguanil (1944), amodiaquine (1946), primaquine (1950), and pyrimethamine (1952), all of which are still being used today. In vitro cultivation did not play any important role in drug screening and development programs until the post-war era.

As part of the antimalarial drug research program during World War II, which was focused on the development of synthetic drugs to replace quinine, fundamental studies to understand the metabolic needs of the malaria parasites cultivated in vitro were undertaken. A rational approach, as opposed to empirical studies, based on the understanding of the biochemistry of malaria parasites, was required for an optimal in vitro culture system. It was during this time that William Trager, who would later head one of the two American research teams that succeeded in developing the continuous in vitro cultivation method, had initiated in vitro studies using *Plasmodium lophurae*, avian malaria parasites that infect chicks and ducks, as the experimental model [22]. A more detailed history of early attempts to cultivate malaria parasites between 1880 and 1946 has been reviewed elsewhere [23,24].

Between 1946 and 1976, a number of other scientists worked independently to develop in vitro cultivation methods for avian, simian, and human malaria parasites. Most employed the rocker dilution technique, rocker perfusion technique, or their variations. All of these efforts had, at best, resulted in short-term cultivation of various *Plasmodium* species. More success was achieved with continuous cultivation of the exoerythrocytic (reticuloendothelial) stages of avian malaria. For the blood stages in human malaria, the only pathogenic stage and the most important focus for chemotherapy, the “right combination” of synthetic tissue culture media and their supplements, buffers, gas mixtures, and other factors that were necessary for the long-term continuous culture were either not available at that time (e.g., production of synthetic culture media at a commercial scale) or not yet discovered. Nevertheless, short-term cultures were useful tools to gain insight into the metabolic needs of malaria parasites and study the mechanisms of action of antimalarial drugs. The technical problems encountered in the early culture systems, described in earlier reviews [15,25], were not resolved until the advent of the new era for experimental research in malaria starting in 1976.

The major breakthrough for the long-term in vitro cultivation of blood stages of *P. falciparum* was attained by two American research teams from Walter Reed Army Institute of Research (WRAIR; Washington, D.C. (currently in Silver Spring, Maryland)) and Rockefeller University (New York) that published their techniques for the long-term in vitro cultivation of *P. falciparum* in 1976 [26,27]. These two methods are based on similar culture conditions (Table 1). The following basic requirements for successful in vitro cultivation are derived from their works: appropriate culture media with serum supplement and adequate buffer, a thin layer of settled human erythrocytes, regular (i.e., daily or at least every other day) change in culture media, addition of fresh, uninfected human erythrocytes at intervals of 2–4 days, and high CO_2_/low O_2_ content. Current methods of in vitro cultivation of *P. falciparum* are essentially based on their methods. As in many scientific works, the long-term cultivation methods were made possible due to many other preceding works (including the early works of William Trager) and may be considered to be a culmination of scientific advances contributed by many scientists in various fields.

The works of the two American research teams on *P. falciparum* led to the first successful continuous in vitro culture of the blood-stage of any *Plasmodium* species. A few years after the publication of these works, the techniques developed for *P. falciparum* were successfully adopted to maintain in vitro the intraerythrocytic stages of simian malarial species, including *Plasmodium knowlesi*, *Plasmodium fragile*, *Plasmodium inui*, *Plasmodium cynomolgi*, and *Plasmodium gonderi* [28,29,30,31,32,33,34,35]. In these early but successful attempts to cultivate simian parasites, such as *P. knowlesi*, rhesus monkey erythrocytes were used [29,32]. Even in more recent attempts to ameliorate the in vitro cultivation of *P. cynomolgi*, monkey erythrocytes and monkey serum (or serum substitutes, including fetal bovine serum, normal horse serum, and Albumax II^™^) were used in a modified protocol based on Trager and Jensen’s cultivation method for *P. falciparum* [36,37]. Quite recently, a British research group succeeded in adapting a *P. knowlesi* strain entirely to continuous culture in human erythrocytes [38]. However, for the three other human *Plasmodium* spp. (i.e., *P. vivax*, *P. malariae*, and *Plasmodium ovale*), limited progress has been made despite various attempts to propagate these parasites indefinitely. It is in this context that the technical capacity to propagate in vitro the asexual intraerythrocytic stages of simian malaria parasites, in particular *P. cynomolgi*, is important for research purposes, as they may serve as a model for non-*P. falciparum* human malaria parasites.

In the following sections, the various factors and conditions required for successful continuous cultivation of *P. falciparum* are reviewed, with an emphasis on the principles of in vitro cultivation. Cultivation techniques of stages other than the intraerythrocytic stages and malaria parasites that infect non-human hosts under natural conditions lie beyond the scope of this review. Readers requiring detailed laboratory protocols on culture procedure, cryopreservation, cloning, and synchronization should consult laboratory manuals and other review papers [39,40,41,42,43,44,45].

## 3. Culture Media and Nutritional Requirements

During the history of the development of eukaryotic cell culture, many types of artificial media and balanced salt solutions have been formulated. Some media have been specifically developed for mammalian cells, while others have been prepared for insect or plant cells. A medium may have been initially formulated to satisfy the nutritional requirements of a particular type of cells, but subsequent studies have shown that it may also support the growth of other types of cells. Despite the plethora of media formulated, developed, and tested over the past 70 years, a large majority of eukaryotic cells are cultivated today in one of the following eight media: basal medium Eagle (BME), Dulbecco’s modified Eagle’s medium (DMEM), Iscove’s modified Dulbecco’s medium (IMDM), McCoy’s 5A medium, medium 199 (M-199), minimum essential medium (MEM), nutrient mixture F-12 Ham, and RPMI 1640. The formulations of these common media were developed and published between 1955 and 1967. These products have become standard media for eukaryotic cell culture and are available from many commercial suppliers.

### 3.1. RPMI 1640

In many cases, the medium of choice for a given type of eukaryotic cell has been determined through numerous empirical trials. For established cell lines, the general rule is to pursue cultivation procedures using the same medium unless there are reasons to change the type of medium. RPMI 1640 is the most widely used culture medium for *P. falciparum* and is the medium of choice since the development of continuous cultivation of this *Plasmodium* species by Trager and Jensen [27]. RPMI 1640 was not “custom-made” to optimize the in vitro growth of *P. falciparum* based on its nutritional requirements. William Trager reasoned that RPMI 1640 may be suitable for maintaining *P. falciparum* in vitro based on the observation that this medium was specifically developed for the cultivation of human leukocytes [46]. In fact, RPMI 1640, usually supplemented with serum from human or animal sources, has wide applications for maintaining the growth of different cell cultures, including parasites other than *P. falciparum*. As in other culture media, RPMI 1640 consists of d-glucose, amino acids, inorganic salts, vitamins, and glutathione (the principal reducing agent in erythrocytes that protects hemoglobin from oxidation), which are essential nutrients required by eukaryotic cells. Mature, enucleated, uninfected human red blood cells maintain functional biosynthetic pathways, ion transport, and buffer systems, including when they are placed in in vitro culture conditions in RPMI 1640 medium [47,48]. The importance of the major components of the culture medium is presented below.

#### 3.1.1. Glucose

Most animal cell lines require medium changes once or twice a week, depending on the growth rate and cell density. For malaria cultivation, daily medium change is the norm. The frequency of medium change may have to be increased to twice or thrice a day for special experiments in which a high yield of parasites (therefore, high parasitemias and/or high hematocrit) is required. Conversely, the frequency of medium change may be reduced to once every 2 or 3 days under certain conditions by lowering hematocrit or starting parasitemia, increasing the buffering capacity or glucose concentration, adding “cold” hypoxanthine (i.e., a naturally occurring purine derivative, as distinguished from tritium-labeled hypoxanthine that may be used as an indicator of parasite growth in drug sensitivity assays [49,50]), or the combination of these factors (temperature can also be reduced).

Glucose is the primary source of energy for most cells. Some investigators have attempted to improve the growth of parasites by increasing d-glucose concentration and adding supplements to RPMI 1640. In one study, in which the medium was changed daily (10% erythrocyte suspension and 0.8–1.5% starting parasitemia), the addition of 2 g/L of extra glucose (i.e., a total of 4 g/L of glucose, including glucose already present in RPMI 1640), reduced glutathione (600 µg/mL) and hypoxanthine (50 µg/mL) to the standard RPMI 1640 buffered with 40 mM (9.15 g/L) TES (RPMI 1640–TES–NaHCO_3_ medium), resulted in a three-fold increase in parasite growth over 4 days in one reference clone compared to the growth in the standard RPMI 1640–HEPES–NaHCO_3_ medium (note: increased glucose needs to be balanced by better buffering or more frequent medium change because of the greater production of lactate) [51]. The growth rate of three other uncloned Thai isolates was not affected by hypoxanthine and glutathione supplements. In another study, it has been shown that laboratory-adapted *P. falciparum* strains (0.5% initial parasitemia, 5% hematocrit, and 5% O_2_–5% CO_2_–90% N_2_ gas mixture) can be cultivated without medium change for three days using a similar glucose-enriched (4 g/L) RPMI 1640–TES–NaHCO_3_ mixture without hypoxanthine supplement [52]. A slight decrease in pH (from 7.4 to 7.1) after three days was not detrimental to parasite growth. This observation was confirmed in another study in which a modified cultivation technique in agitated culture flasks (1% hematocrit and 1% initial parasitemia) with high glucose (4 g/L) concentration and a TES buffer (32 mM) in RPMI 1640 allowed a high yield of parasites without changing the culture medium for up to 3 days [53]. Ofulla et al. also reported that the addition of an extra 2 g/L of glucose increases growth rates in short-term cultures [54].

For routine cultures with daily medium changes, hematocrit and initial parasitemia should be adjusted and kept relatively low. The same principle applies to in vitro drug susceptibility assays in which the medium is not changed for 24–72 h. This is also important from the point of view of the inoculum effect on 50% inhibitory concentration (IC_50_) endpoints in drug susceptibility assays. The concentrations of glucose and buffer do not need to be increased for most experiments and assays. It should be kept in mind that the addition of high concentrations of glucose to the culture medium may result in a hypertonic solution (normal osmolarity in human plasma, 290 mOsM/kg), which is deleterious for parasite growth. For routine cultivation of *P. falciparum* parasites maintained at relatively low parasitemia (< 5%), d-glucose contained in RPMI 1640 is sufficient for optimal parasite growth if the culture medium is changed daily.

#### 3.1.2. Amino Acids

The most thorough analysis of exogenous amino acid and vitamin requirement of *P. falciparum* maintained in vitro was performed by Divo et al. [55]. In their study, an RPMI 1640 medium containing hypoxanthine was entirely reconstituted, and parasite growth of culture-adapted *P. falciparum* strains in reconstituted RPMI 1640 media (supplemented with dialyzed human serum) without one amino acid or one vitamin was assessed over 48 to 96 h and compared with a reconstituted RPMI 1640 medium with a complete set of amino acids and vitamins. Dialyzed human serum lacks small water-soluble molecules (including amino acids, peptides, vitamins, purines) but retains macromolecules, such as proteins. When extensively dialyzed, human serum does not support the growth of parasites unless it is supplemented with hypoxanthine. The study demonstrated that exogenous amino acids in the culture medium are essential for parasite growth, implying that other potential sources of amino acids, including hemoglobin degradation by the parasites and serum, are not sufficient to sustain optimal parasite growth. Among 20 amino acids present in RPMI 1640, isoleucine and methionine are the most important for short-term cultures of laboratory-adapted *P. falciparum* strains. The absence of one of these two amino acids would result in a marked decrease (i.e., >75%) in parasite growth after 96 h. The absence of cystine, glutamate, glutamine, proline, or tyrosine would also lead to growth inhibition, but to a much lesser extent. Deletion of other amino acids has no apparent effect on parasite growth over 96 h.

A series of “follow-up” in vitro experiments confirmed that RPMI 1640 with isoleucine (20–147.5 µM), without any other amino acids, supported the growth of a reference clone (3D7) in long-term continuous cultivation (>2 months) to the same extent as the standard RPMI 1640 medium containing all 20 amino acids [56]. The parasite seems to be dependent on an exogenous supply of isoleucine because this amino acid is absent from human hemoglobin. An additional requirement for exogenous methionine (present at a low level in hemoglobin) may be strain-dependent.

The RPMI 1640 medium, as well as other synthetic culture media, contains amino acids at much higher concentrations than in human plasma [57]. High concentrations of amino acids have been thought by some malaria researchers to be beneficial for sustaining high parasite multiplication rates in vitro. Others have questioned whether malaria parasites actually require an exogenous source of amino acids. The asexual parasites ingest erythrocyte cytosol and catabolize hemoglobin in the food vacuole, where several enzymes (i.e., aspartic proteases (plasmepsins) and cysteine proteases (falcipains)) necessary to digest globin are found [58]. Based on these observations, it has been suggested that hemoglobin digestion may be the major source of amino acids for the erythrocytic stages of malaria parasites [47,48,59].

Malaria parasites can also selectively take up host serum proteins and digest them [60,61]. How the parasites obtain direct access to macromolecules is still not known. Endocytosis through cytostome, tubovesicular membranes, and/or the controversial parasitophorous duct may be involved [62,63,64,65,66]. The currently available data are not adequate to determine the role and contribution of exogenous (RPMI 1640 medium for in vitro cultivation) and endogenous (human plasma and hemoglobin in vivo and in vitro cultivation) sources of amino acids. It has also been suggested that *P. falciparum* may maintain several sources and pathways, including multiple proteases with overlapping functions in its food vacuole (plasmepsins and falcipains), to “guarantee” the supply of essential amino acids [56].

It may be questioned whether parasite physiology, in particular its basal metabolism, can be studied when the parasites are placed in an artificial environment with high amino acid concentrations, whether the medium is compatible with the identification of candidate biochemical targets and drug screening, and to what extent individual parasites constituting a field or clinical isolate are selected during adaptation to in vitro culture conditions, possibly limiting parasite diversity [57]. For example, an experimental study has suggested that high concentrations of l-cysteine (i.e., l-cysteine in RPMI 1640 plus additional exogenous l-cysteine) may inhibit parasite growth [67]). The significance and implications of that experimental study are not clear at present. Further experiments are clearly needed to define the basic nutritional needs of *P. falciparum* parasites.

#### 3.1.3. Inorganic Salts

The balanced salt solution provides inorganic salts required for basal metabolism at sufficient and balanced concentrations that produce osmotic pressure similar to in vivo conditions. Early studies had shown that an addition of inorganic salts that are not in RPMI 1640, such as FeSO_4_, CuSO_4_, and ZnSO_4_, had no beneficial effect on parasite growth [51]. In sharp contrast to amino acids and vitamins, the ionic composition of RPMI 1640 and similar culture media is within or near the physiological values of human plasma [57]. This is probably due to the assumption that osmoregulation is one of the important mechanisms that characterize a complex organization of mammalian cells, tissues, and organs, to maintain physiological levels of ions, including a narrow range of pH values, for optimal growth.

The intraerythrocytic malaria parasite is thought to modify the ionic composition of the host erythrocyte through an ion channel called “plasmodial surface anion channels (PSAC)”, which is expressed and exported by the parasite to the host erythrocyte membrane [68,69,70,71,72]. The ion channel induces a non-specific permeability, allowing various solutes, such as amino acids, sugars (except for disaccharides), glutathione, purines, pantothenic acid, and anions, but not cations (e.g., Na^+^), to enter and accumulate in the host erythrocyte. However, in vitro cultivation of reference strains of *P. falciparum* in sucrose (impermeant disaccharide)-based RPMI 1640 with modified Na^+^ or K^+^ concentrations and dialyzed human serum suggested that low Na^+^ (as low as 6.8 mM present in the serum supplement, compared to 142.8 mM in the standard RPMI 1640 medium) and high K^+^ concentrations (up to 148 mM, compared to 5.4 mM in the standard RPMI 1640 medium) support parasite growth to a similar extent as the standard medium [73].

Among essential ions that are critically important for malaria parasites, calcium (Ca^2+^) plays various roles to maintain normal cell functions and growth. Ca^2+^ concentration in the cytosol of mature *P. falciparum* trophozoite has been estimated to be 0.1 µM [74]. The parasite accumulates Ca^2+^ (through increased parasite membrane permeability, erythrocyte Ca^2+^ ATPase, and possibly Ca^2+^ channel) and stores Ca^2+^ in its organelles (endoplasmic reticulum, food vacuole, mitochondrion, and/or acidocalcisome) [75,76,77,78]. The mechanisms involved in Ca^2+^ transport in the intraerythrocytic parasite remain poorly understood, and the concentrations of Ca^2+^ in different compartments and organelles are technically difficult to determine.

For Mg^2+^ (0.41 mM in RPMI 1640 and 0.55 mM in human plasma [57]), concentrations between 0.5 and 3 mM have no effect on the in vitro growth of laboratory-adapted *P. falciparum* strains over several asexual intraerythrocytic cycles, but both high Mg^2+^ concentrations (5 mM) and the absence of Mg^2+^ in the medium (i.e., cultivation in Mg^2+^-free RPMI 1640 medium) result in growth retardation by 35–43% after 48 h of cultivation [79].

Further experiments are required to determine the optimal extracellular and intraerythrocytic ionic concentrations that support in vitro parasite growth. Other transport mechanisms or organelles, such as passive diffusion, host erythrocyte channels, cystostome, tubovesicular membranes, and/or the controversial parasitophorous duct, may also possibly be involved in the acquisition of various nutrients and the modification of the host environment [62,63,64,65,66,80].

An early experimental study showed that once the complete RPMI 1640 medium containing 10% human serum (or animal serum) is used to cultivate malaria-infected erythrocytes or even uninfected erythrocytes for up to 12 h and subsequently stored at –20 °C, the spent media do not support parasite growth of the same parasite strain, even when fresh serum (final serum concentration, 20% *v*/*v*) is added [29]. The spent medium incubated with uninfected erythrocytes supports parasite growth better than the spent medium exposed to infected erythrocytes, but not to the extent that fresh medium supports parasite growth. This inhibitory effect is not due to the buffering capacity of the medium since the pH was not altered compared with the fresh medium. The study did not identify which RPMI 1640 component was altered when the culture medium was exposed to the erythrocytes.

It has been shown that any change in the concentrations of inorganic salts, d-glucose (for example, increasing from 2 g/L supplied in the medium to 3 g/L), glutathione, adenosine, and amino acids (such as isoleucine and methionine) leads to either no improvement in parasite growth or, worse, poor parasite growth [55,81]. Despite the fact that there is no specific culture medium designed for malaria cultivation and some modified RPMI 1640 media have been shown to support the growth of laboratory-adapted strains of *P. falciparum* in various experimental studies, it can still be said today, in 2023, that, by and large, among commercially available culture media, in the words of Jensen and Trager [81], “the RPMI 1640 medium is so well balanced that alterations are generally deleterious”. A possible exception to this general rule may be the beneficial effect of “cold” hypoxanthine added to the RPMI 1640 medium, especially when Albumax^®^, instead of human serum, is used to supplement the medium.

#### 3.1.4. Vitamins

Vitamins are present at supraphysiological concentrations in RPMI 1640 [57]. In an early study, pantothenate (also referred to as d-pantothenic acid or vitamin B5, a precursor of coenzyme A) was shown to be the only vitamin necessary for optimal parasite growth for 96 h [55]. Whereas uninfected human erythrocytes are impermeable to pantothenate, *P. falciparum* develops its own pantothenate uptake system through a specific transporter [82,83]. The biosynthetic pathway of the transformation of pantothenate to coenzyme A has been characterized in *P. falciparum* [84]. The dependence of the parasite on an exogenous source of pantothenate is further supported by experiments, which demonstrated the inhibitory effects of pantothenate analogs against a laboratory-adapted *P. falciparum* strain [85].

In the study conducted by Divo et al. [55], the “minimal medium” consisting of 10% dialyzed human serum and the seven amino acids and pantothenate in the “basal medium” (inorganic salts, hypoxanthine, glutathione, HEPES buffer) was successfully used to maintain culture-adapted *P. falciparum* strains for up to 6 weeks. The “basal medium” supplemented with 10% undialyzed human serum and pantothenate but without any additional amino acids supported parasite growth to the level comparable to normal RPMI 1640 supplemented with 10% undialyzed human serum for only 48 h. Despite the major insight into the parasite’s nutritional requirement brought about by the study conducted by Divo et al. [55], the exact role of the erythrocyte stores of vitamins (also amino acids) in supplying nutrients to *P. falciparum* is still not well-known [86]. Further experiments on the modified composition of RPMI 1640 did not lead to any improvement in maintaining the parasites in vitro.

More recent evidence from biochemical and genomic studies suggested that intraerythrocytic *P. falciparum* may have functional vitamin B1 (thiamine) and B6 (pyridoxine) biosynthetic pathways and/or uptake systems [87,88]. However, there seem to be no plasmodial genes that encode enzymes of the biosynthetic pathways of vitamin C, D_3_, E, and biotin [84].

Folic acid (vitamin B9) is one of the molecules collectively referred to as “folates”. Folates play several important roles in cellular functions as a donor of a one-carbon group, including nucleotide (purine) synthesis, synthesis of some amino acids (e.g., methionine), and amino acid metabolism. *Plasmodium falciparum* takes up exogenous folic acid and *para*-aminobenzoic acid (PABA), which are both present in RPMI 1640. Unlike humans, who cannot synthesize folate and require folate in their diet, malaria parasites synthesize folate de novo through intricate pathways that have not yet been fully elucidated [89,90]. Nonetheless, the essential features of the biosynthetic pathway have been known for decades, and two of its key enzymes (note: they are bifunctional enzymes in *P. falciparum*), hydroxymethyldihydropterin pyrophosphokinase-dihydropteroate synthase (DHPS) and dihydrofolate reductase (DHFR)-thymidylate synthase, have been used as drug targets of sulfones/sulfa drugs and DHFR inhibitors (pyrimethamine, cycloguanil (biologically active metabolite of proguanil)), respectively [91].

### 3.2. Alternative Culture Media

Many other culture media contain similar components as RPMI 1640, with the same amino acids and vitamins and similar inorganic salts, usually, but not always, at slightly different concentrations. Some media contain more vitamins and other components, such as cholesterol, fatty acids, purines, and pyrimidines, compared with RPMI 1640. The following are some commercially available media that support the in vitro growth of asexual intraerythrocytic stages of *P. falciparum*: M-199 with either Hank’s or Eagle’s salt solution, Ham’s nutrient mixture F-12, DMEM, IMDM, Waymouth medium, and NCTC 135 (medium formulated at the National Cancer Institute, Tissue Culture section) [26,30,92,93,94,95,96,97,98,99]. In addition to these media, RPMI 1630 was reported to support parasite growth in vitro [96], but it is not available from commercial sources.

The GIT medium (Wako Pure Chemical Industries Ltd., Osaka, Japan) is a 1:1 (*v*/*v*) mixture of IMDM and Ham’s F-12 nutrient medium supplemented with an ammonium sulfate fraction of adult bovine serum [100]. It has been used almost exclusively in Japanese research laboratories for cell cultures, including experimental studies on *P. falciparum* cultivation [101,102]. Outside Japan, GIT was used successfully to perform ex vivo drug susceptibility assays (i.e., using clinical isolates directly for assays without prior adaptation to in vitro culture) and to initiate cultivation of fresh clinical isolates and maintain them in culture for up to 14 days in Cameroon [103].

A single study has shown that in vitro cultivation of fresh clinical isolates of *P. falciparum* can be maintained for several weeks using plant tissue culture media, Nitsch medium, and White’s medium, supplemented with 10% human serum and 2 mg/mL d-glucose and buffered with 25 mM HEPES and 29 mM NaHCO_3_, without prior adaptation to these media [97]. The authors reported that the multiplication rate in these plant tissue culture media was similar to RPMI 1640. Although further experiments are required to validate these results, especially for the long-term cultivation of a panel of reference *P. falciparum* strains beyond four weeks, at present, there seems to be no particular advantage in using plant tissue culture media for the cultivation of *P. falciparum*.

Before 1976, experimental studies on short-term *P. falciparum* culture were generally conducted with the Harvard medium (prepared on the basis of a chemical analysis of monkey plasma; not available from commercial sources), CMRL-1066 (medium developed at the Connaugh Medical Research Laboratories), or M-199, buffered with glycylglycine and/or sodium bicarbonate and supplemented with d-glucose and fetal calf serum or human serum and incubated in 5% CO_2_/95% air [104,105]. Except for M-199, these aforementioned culture media are no longer used for malaria parasite cultivation.

Modified M-199 had been used for the continuous cultivation of *P. falciparum*. In one study [26], M-199 containing Eagle’s salts was supplemented with d-glucose (2 mg/mL), l-glutamine (2 mM), 2-mercaptoethanol (0.3 µM), dl-α-tocopherol (30 µg/mL), and 10% heat-inactivated fetal bovine serum. Cryopreserved parasites were thawed and successfully maintained in continuous culture for more than three weeks. In another study [30], M-199 was supplemented with adenosine triphosphate (ATP), adenosine, inositol, coenzyme A, creatinine, glutathione, glyglycine, d-glucose, vitamin C, 15–20% pooled, and heat-inactivated calf serum to initiate the long-term cultivation of cryopreserved isolates and maintain the strain for over a year. The growth of a laboratory-adapted strain routinely maintained in RPMI 1640 was compared in Ham’s F12, M-199 with Earle’s salts, and M-199 with Hank’s salts after a 96 h incubation [93]. Parasite growth in Ham’s F12 was comparable, or slightly less, with RPMI 1640 when the media were supplemented with 5% or 10% human serum. Parasite growth in M-199 with Earle’s salts or Hank’s salts was two-to-three-fold less than either RPMI 1640 or Ham’s F12. The use of unsupplemented M-199 resulted in suboptimal parasite growth. In short, there seems to be no advantage in using Ham’s F12 or M-199 instead of RPMI 1640 for the continuous culture of *P. falciparum*.

A series of experiments on the short-term ex vivo cultivation of fresh clinical isolates was performed with various commercially prepared culture media [103]. DMEM and IMDM consistently supported the in vitro growth of parasites during the first intraerythrocytic cycle and were usually better than RPMI 1640 in most isolates. However, these media may not be suitable to establish a continuous culture or even for a short-term culture from and beyond the second life cycle. GIT was another medium that supported the growth of fresh isolates for both short-term and long-term cultivation. Unmodified Ham’s nutrient mixtures F-12 and M-199 did not support the in vitro growth of fresh isolates, as well as RPMI 1640.

In other studies on laboratory-adapted strains [98,99], it has been shown that various 1:1 (*v*/*v*) combinations of two of the following media, RPMI 1640, NCTC 135, and IMDM, supplemented with 10% human serum, do not improve parasite growth over the control medium (RPMI 1640 with 10% human serum) during a 7-day cultivation period. A 2:1:1 mixture of three media resulted in about a 50% increase in parasitemia. The media with serum substitutes, such as Albumax^®^ and some animal sera (fetal calf, horse, rabbit, and goat), also supported the short-term culture with prior adaptation. Furthermore, the authors reported that this combination of three media, supplemented with 10% fetal bovine serum or 10% horse serum, may also be suitable for long-term cultivation.

Although parasite growth using these media may be comparable with RPMI 1640 for short-term and/or long-term culture, most do not seem to offer any distinct advantage over the latter medium. The capacity of some other media to support parasite development for a short-term period has been evaluated, but none were comparable to RPMI 1640 [103,106]. Therefore, after more than 45 years of experience with different malaria researchers, RPMI 1640 remains the standard medium of choice for the in vitro culture of *P. falciparum*. Future studies may possibly reveal that serum-free media are useful for routine cultivation of *P. falciparum*, but more short-term and long-term cultivation experiments will be needed to ascertain whether the majority of laboratory-adapted strains and fresh isolates adapt to alternative media.

## 4. Buffer

### 4.1. pH Regulation in the Human Host

Acid-base regulation is one of the key metabolic activities that characterize a living system. Cell metabolism involves the biosynthesis of many substances as well as the excretion of waste by-products that are generally acidic. In the human host, buffer systems, lungs, and kidneys regulate H^+^ concentration. Under physiological conditions, CO_2_, the major metabolic product that yields H_2_CO_3_, and lactic acid produced due to muscular activities are neutralized by buffering substances in the blood to maintain blood pH within a narrow range (normal range, pH 7.36–7.44 or 36–44 nM H^+^). The principal buffers in the blood are the carbonic acid/bicarbonate system (H_2_CO_3_/HCO_3_^−^) in plasma, hemoglobin, and plasma proteins. The bicarbonate buffer system is the most important pH regulatory system in the extracellular compartment. An increased H^+^ concentration in the extracellular fluid is buffered by HCO_3_^−^, resulting in increased CO_2_ and H_2_O production. Increased OH^−^ concentration in the extracellular fluid results in a reaction with H_2_CO_3_ to form HCO_3_^−^. Excessive CO_2_ and HCO_3_^−^ are eliminated by the lungs and kidneys, respectively. The bicarbonate buffer system regulates the pH of the extracellular fluid and is jointly coordinated by the lungs and the kidneys. The protein component of hemoglobin and plasma proteins function as bases (i.e., proton acceptors) due to some amino acids with negative charges. The buffering capacity of hemoglobin is as important as the bicarbonate buffer system. The plasma protein buffer system plays a limited role. There are other buffer systems in the human body, such as the phosphate buffer system, but they do not have a direct bearing on in vitro cell culture.

### 4.2. Buffers in Malaria Cultivation

In a closed in vitro system in which the medium is not continuously replaced, acidic by-products accumulate rapidly in the extracellular environment and decrease the pH of the medium unless a buffer system neutralizes H^+^, and the spent medium is removed and replaced with a fresh medium regularly. The combination of NaHCO_3_ added to RPMI 1640 medium, erythrocytes, plasma (or serum) supplement, and gaseous CO_2_ (usually 5% in a CO_2_ incubator, 3% in a candle jar versus 5–6% CO_2_ in human arterial and venous blood), simulates the in vivo conditions.

Sodium bicarbonate is used as one of the buffering systems in the method developed by Trager and Jensen [27]. In fact, most types of cells are cultivated using the bicarbonate/CO_2_ buffering system. This system was originally designed to maintain the pH of culture media within the physiological range in gas mixtures containing 5% CO_2_ and 95% air. Carbon dioxide gas dissolves in liquid media and affects pH (CO_2_ + H_2_O ↔ H^+^ + HCO_3_^−^). At a constant NaHCO_3_ concentration, a lower percentage of CO_2_ (1–4%) in the gas mixture increases pH [107]. Increasing or decreasing the bicarbonate concentration results in lower rates of parasite growth [54].

Since the in vitro culture of many types of cells is performed in 5% CO_2_, the addition of NaHCO_3_ to commercial media has become a standard practice today. The use of NaHCO_3_ is appropriate for malaria cultures in a candle jar (approximately 3% CO_2_ and 17–18% O_2_) or in a 5% CO_2_ incubator. The main disadvantage of bicarbonate is suboptimal buffering capacity within the range of normal physiological pH due to its pKa of 6.3 at 37 °C. In addition to sodium bicarbonate, most culture media, including RPMI 1640, contain phosphate salts, which act as buffers. However, NaHCO_3_/gaseous CO_2_ systems, phosphates, erythrocytes (hemoglobin), and plasma proteins have a limited buffering capacity to maintain the pH of malaria cultures beyond 24 h, especially at high (>1%) parasitemias.

During the early development of cultivation techniques, one of the major technical challenges of developing cultivation methods for malaria parasites was the maintenance of the pH of the medium within a narrow range of about 7.3–7.5. Two technical solutions were possible to maintain the physiological pH: (i) a perfusion or continuous flow culture system that constantly removes lactic acid and adds fresh culture medium and (ii) the supplementation of the culture medium with zwitterion buffers in dilution systems. Perfusion or flow systems require sophisticated devices and are not suitable for field use. The use of zwitterionic buffers is a practical solution that is applicable even in field laboratories.

The choice of buffers was found to be one of the keys to successful cultivation. Zwitterions have both positive (secondary and tertiary amino groups) and negative (sulfonic and carboxylic groups) ions within the same molecule. Zwitterionic buffers were introduced toward the late-1960s. The buffers that were evaluated for in vitro cultivation of malaria parasites in earlier studies include N-glycylglycine (pKa 7.9 at 37 °C), N,N′-bis(2-hydroxyethyl)-2-aminoethanesulfonic acid (BES; pKa 7.1 at 25 °C, 6.9 at 37 °C), N-tris(hydroxymethyl)methyl-2-aminoethanesulfonic acid (TES; pKa 7.4 at 25 °C, 7.2 at 37 °C), and N-2-hydroxyethylpiperazine-N′-2-ethanesulfonic acid (HEPES; pKa 7.5 at 25 °C, 7.3 at 37 °C) [108]. The use of TES and HEPES is associated with the improved buffering capacity of the culture medium and increased parasite growth. These two buffers are characterized by similar useful pH ranges (6.8–8.2) and pKa values closer to the useful pH range for cell culture media. Haynes et al. [26] and Trager and Jensen [27] have adopted TES and HEPES for their cultivation techniques, respectively. Zwitterions provide an additional advantage of being minimally influenced by changes in the gas phase, maintaining the pH of the medium when cultures are placed in normal air, i.e., outside the CO_2_ incubator or candle jar, during experiments, or a manual change in the culture medium.

Most, if not all, researchers today use HEPES, instead of TES, in addition to NaHCO_3_ as buffers for *P. falciparum* cultivation. The buffering capacity of HEPES and TES for malaria cultivation has not been compared in many studies. In one study, the multiplication rate of the reference strain FCR-3 was higher at the end of a 72 h cultivation period in the RPMI 1640 medium buffered with 25 mM HEPES and supplemented with glucose (4 g/L) and hypoxanthine (44 mg/L) compared to the multiplication rate in the medium buffered with 40 mM TES and supplemented with glucose (2 g/L) [109]. However, it is not clear whether this difference was due to the seemingly higher concentration of glucose provided in the RPMI 1640 medium buffered with HEPES and the absence of hypoxanthine supplement in the RPMI 1640 medium buffered with TES in this experiment. Since there seems to be no advantage to using TES instead of HEPES for malaria cultivation, the latter seems to be the best choice.

In addition to the choice of buffers, the number of parasites in relation to the volume of the culture medium should be kept low to limit the accumulation of lactic acid. Buffers in the culture medium can maintain neutral pH over 24 h, and even for one complete erythrocytic cycle of *P. falciparum*, provided that hematocrit (usually <12%, preferably much lower) and initial parasitemia (usually <1%) are kept low. Cultivation at higher hematocrit and/or parasitemia is possible by either increasing the frequency of medium change (i.e., instead of once daily, increase to twice or thrice daily), increasing the buffering capacity of the medium, or using special cultivation devices.

Cultures that are growing well show high glucose consumption by the parasites and the accumulation of high lactic acid concentrations in the extracellular medium (two moles of lactate per mole glucose), resulting in decreasing pH over time. Depending on the developmental stage of asexual parasites, infected erythrocytes may produce 5–100 times more lactic acid than uninfected erythrocytes [110]. Trophozoites and schizonts excrete 2–20 times more lactic acid than rings [110,111]. Optimal parasite growth occurs when the parasites are maintained at relatively low parasitemias (<2.5%) with extracellular lactic acid concentration <12 mM and the medium (RPMI 1640–40 mM TES–NaHCO_3_ with 50 µg/mL hypoxanthine and a total of 4 g/L glucose) is changed every 12 h [110]. At an extracellular lactic acid concentration of 12–25 mM, more frequent medium changes and/or the diminution of parasitemia are necessary to avoid growth inhibition. When the parasites are exposed to extracellular lactic acid concentrations >25 mM for more than 6 h, irreversible damage and parasite death occur.

In Trager and Jensen’s culture method, buffering with 25 mM HEPES seems to be optimal for the routine maintenance of cultures. Increasing the concentration to 50 mM HEPES, with or without a 50% decrease in NaHCO_3_ concentration, does not improve parasite growth [81]. However, the addition of 35 mM HEPES, instead of 25 mM, and a reduction in hematocrit may allow cultivation without medium change for up to 3 days [112,113]. At 37 °C, the pKa values of NaHCO_3_ and HEPES are 6.3 and 7.3, respectively. The combined HEPES-NaHCO_3_ buffer system brings the buffering range close to the optimal physiological pH range (7.2–7.4).

## 5. Erythrocytes

Human erythrocytes are required for optimal in vitro growth of *P. falciparum*. Unless the complete life cycle is being initiated experimentally from sporozoites, the usual source of asexual erythrocytic *P. falciparum* culture is previously infected erythrocytes that have been cryopreserved, passaged in suitable animal hosts, or freshly obtained from patients. The most convenient and usual procedure, especially in the field, is to use the patients’ own infected erythrocytes to initiate in vitro culture. If the initial parasitemia is less than 1%, the patient’s infected erythrocytes can be directly used for culture. If the initial parasitemia is >1% and cultivation is performed in the field, it is recommended to dilute the infected erythrocytes with uninfected erythrocytes from a donor who has not taken an antimalarial drug (or antibiotics with antimalarial activity) in the recent past. To maintain the parasites in vitro beyond the first or second intraerythrocytic cycle, a regular addition of uninfected human erythrocytes, at least once every two intraerythrocytic cycles (i.e., every 4 days), is required.

The ability of fresh isolates of *P. falciparum* to adapt to in vitro cultivation, as well as the growth rates, is variable [114]. Several factors may explain these variations, including initial parasitemia, frequency, and the extent of red cell dilution, nutritional qualities of serum, and recent exposure to drug treatment before cultivation. In the experience of Trager and Jensen [115], the major factor underlying these variations is the still undefined innate characteristic of the donor’s erythrocytes to support parasite growth. The results of a recent study tend to support the intuitive conclusion of Trager and Jensen. The multiplication rate of the 3D7 clone varied from 64% to 136% (compared to the mean multiplication rate in normal erythrocytes set to 100%) in different samples of erythrocytes of healthy blood donors with recent African ancestry without any blood disorders (e.g., hemoglobin S, hemoglobin C, thalassemia, glucose-6-phosphate dehydrogenase (G6PD) deficiency, hereditary elliptocytosis) [116]. Parasites grew at a higher multiplication rate over 48 h in the erythrocytes with higher membrane deformability, larger mean volume (i.e., mean corpuscular volume, MCV), a higher mass of hemoglobin per cell (i.e., mean corpuscular hemoglobin, MCH), and less fragile membranes. The genotypic profiles of the erythrocytes that predict a higher multiplication rate of *P. falciparum* have not been established in that study, but may include polymorphisms in ion channel proteins, red blood cell membrane backbone, and/or red cell plasma membrane proteins (which include receptors required for merozoite invasion).

### 5.1. Sources of Uninfected Erythrocytes and Anticoagulants

In the late-1970s and early-1980s, some researchers used erythrocytes from simian sources to cultivate *P. falciparum* for experimental purposes. Haynes et al. [26] initiated the continuous in vitro culture from a cryopreserved, chimpanzee-adapted *P. falciparum* strain in erythrocytes from chimpanzees (*Pan troglodytes*), owl monkeys (*Aotus trivirgatus*), and humans. This parasite strain failed to develop in erythrocytes from rhesus monkeys (*Macaca mulatta*) and guinea pigs. Peterson et al. [117] also successfully cultivated several strains of *P. falciparum* in the erythrocytes of *Aotus trivirgatus*. Attempts to propagate *P. falciparum* strains in vitro using *Saimiri sciureus* (squirrel monkey) erythrocytes were met with limited success, i.e., up to three weeks of continuous culture [118]. By contrast, few laboratory-adapted *P. falciparum* strains (Palo Alto I/Uganda, FCB/Colombia) have been adapted to propagate in vivo in splenectomized *Saimiri sciureus* and adapted back to in vitro propagation in human erythrocytes [119,120]. Except for specific experimental purposes, human erythrocytes are used for malaria cultivation today.

The principal source of uninfected human erythrocytes, kept at +4 °C, is blood banks. Several blood storage protocols have been developed with different preservatives added at the time of blood collection from healthy donors. Blood preservatives are usually predosed in an aseptic collection pouch so that venous blood being collected is immediately mixed with the preservative. Blood preservatives that have been or are commonly used at blood banks include acid–citrate–dextrose (ACD), citrate–phosphate–dextrose (CPD) modified CPD with 0.2 g/L adenine (CPD-A), and saline–adenine–glucose (SAG; or saline–adenine–glucose–mannitol, SAGM). CPD or CPD-A is the most commonly used anticoagulant. Judging from transfusion malaria occurrences, *P. falciparum*-infected cells in CPD retain their infectivity well in the refrigerator. Whole blood may be initially collected on CPD and centrifuged, and their plasma may be removed for other medical uses. The remaining blood is stored as packed cells.

In an early study, Jensen and Trager recommended the use of uninfected erythrocytes stored at +4 °C in citrated anticoagulants (ACD or CPD) for 2 to 4 weeks, rather than using erythrocytes freshly drawn from donors, to obtain an optimal growth of culture-adapted laboratory strains [81]. In a later study, it was reported that fresh blood collected on CPD and blood stored in CPD for 16–20 days resulted in up to a 2.3-fold increase in parasite growth compared with blood stored for 4–16 days [121]. The type of anticoagulants (CPD, CPD-A, and protein-poor preservatives) did not influence parasite growth when blood was stored for up to 30 days. Erythrocytes stored as packed cells were not suitable for culture beyond 21 days. These authors also noted that erythrocytes stored in CPD and protein-poor preservatives containing adenine supported parasite growth even after 84 days. In practice, most malaria research laboratories procure donors’ blood on a monthly basis so that any leftover blood beyond five weeks is discarded. In a more recent study, it was reported that high parasitemia and high multiplication rates of laboratory-adapted *P. falciparum* strains were consistently attained when erythrocytes stored at 4 °C in CPDA (as whole blood) for less than two weeks were used in cultures [122]. Another recent study confirmed that the growth of reference strains of *P. falciparum* was comparable in fresh erythrocytes collected into ACD anticoagulant and those that were stored as packed erythrocytes at 4 °C for two weeks, but was significantly diminished in erythrocytes stored in the same condition (packed erythrocytes at 4 °C) for four weeks (35–58% growth) and six weeks (<10% growth) [123]. Similar results were obtained with different storage buffers and anticoagulants, including ACD and CPDA. Contrary to some of the aforementioned early studies [81,121], a South Korean research group reported that, compared to the growth rate in freshly collected erythrocytes, the 96 h multiplication rates of a reference strain (FCR-3) cultivated under standard conditions (RPMI 1640 supplemented with 10% type AB^+^ human serum; 5% hematocrit; starting parasitemia 0.1% in suspension culture with constant shaking, in 5% CO_2_, 5% O_2_, and 90% N_2_, and medium change every 12 h) decreased as the age of stored erythrocytes increased from 1 to 4 weeks [124]. The final parasitemias in cultures with 4-week-old erythrocytes collected in ACD or EDTA were 70% and 50% of those attained with freshly collected erythrocytes, respectively. The underlying reasons for these apparently discordant results have not been explored, but they may be partly due to the short cultivation period (i.e., two cycles) in the latter study.

From a practical viewpoint, fresh erythrocytes are usually not available on a daily or weekly basis in most malaria research laboratories. Although it would be ideal if a new batch of fresh erythrocytes could be obtained every two weeks [122], erythrocytes stored for up to 4 weeks are suitable for routine cultivation procedures. For optimal storage, erythrocytes should be kept in plasma until use. Erythrocytes are washed with RPMI 1640 before use.

Parasite growth was also reported to be comparable in erythrocytes from whole blood collected on CPD-A, packed cells stored in CPD-A, and packed cells stored in SAGM preservative (plasma from a 500 mL donor’s blood is removed and replaced with 100 mL of SAGM) [125]. Likewise, the use of fresh erythrocytes or erythrocytes stored in CPD for 4 weeks before use resulted in similar parasite growth during the 14-day cultivation period of a laboratory-adapted strain [29]. However, hemolysis tends to occur in red cells stored for over 5 weeks. Such outdated erythrocytes are not suitable for cultures. When the growth of several culture-adapted *P. falciparum* strains was compared over more than 1 month in the uninfected erythrocytes stored as whole blood in CPD and those stored as packed red cells without plasma in SAG, higher parasitemia was consistently observed with the erythrocytes stored in SAG [51].

An unconventional source of uninfected human blood for *P. falciparum* cultivation is biopreserved erythrocytes. It was reported that parasite growth in biopreserved erythrocytes was comparable to fresh erythrocytes [123]. For cryopreservation, fresh erythrocytes mixed with human plasma were added to freezing media (28% glycerol, 3% sorbitol, 0.65% NaCl). Biopreserved erythrocytes have not been used extensively for malaria cultivation but may be useful for experimental purposes.

Another source of human erythrocytes is human hematopoietic stem cells [126]. Culture systems of various protozoans, including *Plasmodium* spp., based on induced pluripotent stem cells are opening up new horizons [127]. One of the applications of human erythrocytes derived from pluripotent stem cells is humanized mice, which may hold promise in different research areas [128,129,130,131,132]. There is hope that this new experimental model will replace non-human primate models. Hematopoietic stem cells can also be used to study parasite biology in future in vitro experiments [133].

### 5.2. Blood Types

In the initial attempts to develop continuous cultivation of *P. falciparum*, malaria-infected erythrocytes of *Aotus* monkeys were mixed with human-type AB^+^ erythrocytes and diluted in an RPMI 1640 medium containing 15% human-type AB^+^ serum to avoid incompatibility with the *Aotus* blood [27]. After several cycles, established cultures were maintained in human-type A^+^ erythrocytes and serum due to their wide availability. Type A^+^ serum is compatible with both types A and O erythrocytes, which represent the dominant blood types in a large majority of Caucasian populations.

Early studies had established that all four human blood groups are suitable for culture [29,134]. If type AB human serum is used to supplement the RPMI 1640 medium, erythrocytes belonging to any of the four blood groups are compatible. This approach is convenient for ex vivo drug susceptibility assays on fresh isolates and the initial cultivation of clinical isolates in the field. Regardless of the initial blood type in which the parasites were present in the original human host, the subsequent in vitro intraerythrocytic cycle can be maintained in any blood type.

It has been hypothesized that *P. falciparum*-infected humans with group O^+^ have a survival advantage and that this selection pressure exerted by *P. falciparum* during the evolutionary history of *Homo sapiens* may explain the current global distribution of the ABO blood group, notably the high frequency of the group O^+^ in sub-Saharan Africa and South America [135]. Several studies have also suggested that type O^+^ is associated with a decreased risk of severe malaria [136,137]. One of the possible implications of this hypothesis is that *P. falciparum* may have adapted better to type O^+^ erythrocytes compared to other blood types. The results of a recent experimental study tend to support this viewpoint. Several laboratory strains of *P. falciparum* incubated simultaneously in the presence of a mixture of A^+^, B^+^, O^+^, and AB^+^ erythrocytes showed a preference for O^+^ erythrocytes [138]. In another experiment, the parasitemias of two reference strains attained after two cycles when cultivated separately in A^+^, B^+^, and O^+^ erythrocytes were higher in O^+^ erythrocytes [139]. There was only a slight (but statistically significant) preference for O^+^ erythrocytes, and the parasites also invaded erythrocytes of the other blood types at lower proportions or parasitemia.

In practice, many malaria research laboratories use type O^+^ and/or A^+^ erythrocytes, which are the most common (i.e., close to ¾ of the human population) blood groups in the world. There seems to be no compelling reason at present to opt for a specific blood type. Any available ABO blood types can be used for *P. falciparum* in vitro cultivation. However, the use of erythrocytes from donors with known hemoglobinopathy (thalassemia, hemoglobin C, sickle cell anemia) should be avoided because *P. falciparum* growth may be suboptimal [116,139,140,141,142,143,144,145,146].

### 5.3. Hematocrit

For continuous culture, parasites are usually maintained in 8–12% erythrocyte suspension (i.e., hematocrit). According to Jensen and Trager [81], 10–12% erythrocyte suspension in a complete RPMI 1640 medium yielding a depth of 3.5 to 4.5 mm in a 35 mm diameter Petri dish results in optimal parasite growth (note: based on the total volume of 1.5 mL of the RPMI human serum mixture with 10% erythrocyte suspension in the Petri dish, the hematocrit layer depth would be approximately 0.16 mm). In addition to the depth of the culture medium, the surface area of the medium is probably just as important to optimize gas exchange between the medium (i.e., O_2_ and CO_2_ dissolved in the medium) and the atmosphere in the incubator.

Depending on the experimental purpose, cultures may be maintained at a lower hematocrit, which favors parasite growth due to the relatively more abundant nutrients and buffering capacity in relation to the absolute number of parasites in Petri dishes, flasks, or culture plates, provided that the parasitemia is also kept at low levels (<2–3%) and fresh medium is added daily. In such conditions (low starting parasitemia of 0.1% and about 5% hematocrit), a more frequent medium change (i.e., the interval of medium change every 12 h versus every 24 h) does not improve the parasite’s multiplication rate over two cycles, although a prolonged delay in medium change (i.e., every 48 h) would result in decreased multiplication rate during the second cycle [124]. In the protocol developed by Radfar et al. to optimize the yield of synchronized reference clones of *P. falciparum* (3D7 and Dd2; RPMI 1640 buffered with 25 mM HEPES and 1.77 mM NaHCO_3_ and supplemented with 0.5% Albumax^®^ I and 100 µM hypoxanthine; 5% CO_2_ in air or 3% CO_2_-1% O_2_-96% N_2_ gas mixture) [122], the authors recommend hematocrit values within the range of 0.8 to 1.5% and proposed a mathematical formula to calculate the approximate volume of the culture medium necessary to maintain parasite growth for 24 h (i.e., until the next change in the medium). Using this protocol (and fresh human erythrocytes stored <14 days), the authors reported that high parasitemia (20–30%) can be obtained in 150 cm^2^ culture flasks (maximum volume of culture medium, 140 mL). For in vitro drug susceptibility assays that require a 42–72 h incubation, cultures in 96-well plates are performed at low hematocrit, generally at 1.5%, to avoid the labor-intensive task of removing the spent medium and adding the fresh medium after the initial 24 h of incubation. The cultures can also be maintained at a much higher hematocrit in special devices. This will be discussed in another section.

Theoretically, parasite growth is optimal at the interface between the culture medium and the uppermost layer of erythrocytes, where the exchange of gas, nutrients, and waste products occurs freely. By contrast, the exchange is suboptimal at the bottom layer of the erythrocytes. Depending on the protocols of cultivation in different research laboratories, the depth of the hematocrit layer may vary from 0.15 mm to 0.8 mm or more. Mathematical models that attempt to describe the complex interplay between parasite multiplication rate, hematocrit layer depth, the width or diameter of culture devices, and various other factors have been developed, and the effects of the dimensions of the hematocrit layer were analyzed to determine the conditions leading to optimal parasite growth [147,148]. One of the key findings using the model and experimental data obtained from *P. falciparum* (3D7 reference strain) cultivated under standard conditions (RPMI 1640 supplemented with 10% human serum, 5% hematocrit, initial parasitemia 0.5%, 5% O_2_, 5% CO_2_, and 90% N_2_) in 6-well plates (35 mm diameter wells) and the renewal of culture medium and dilution of cultures to 0.5% parasitemia every 48 h was that optimal development (i.e., highest parasitemia after 48 h) occurs in a hematocrit layer depth between 0.18 mm and 0.34 mm. This value is close to the theoretical hematocrit layer depth found empirically by Jensen and Trager (i.e., approximately 0.16 mm) [81]. Parasite growth diminished considerably when the depth of the hematocrit layer exceeded 1 mm.

### 5.4. Leukocytes

Leukocytes are present in whole blood or packed red blood cells obtained from blood banks or in whole blood collected directly from patients. An early study showed that leukocytes from Gambian children with acute malaria inhibit parasite growth [149]. In another more recent study conducted in Tanzania, blood samples were obtained from malaria-infected children <5 years old, and the isolates were cultivated in vitro after the removal of leukocytes by the filtration of venous blood through the cellulose column [150]. Compared to the control isolates (i.e., unfiltered blood samples), parasites in cellulose-filtered samples grew faster and attained higher parasitemias during the first three cycles. Cultivation was not pursued beyond three cycles to assess the long-term beneficial effect of leukocyte-free blood to adapt fresh clinical isolates from African patients to in vitro conditions. By contrast, studies conducted in non-endemic countries demonstrated that the presence of leukocytes, some of which may be intact while others are in the process of disintegration, does not affect in vitro parasite development [121]. These authors reported that the elimination of leukocytes by passage through Whatman CF-11-powdered cellulose results in a lower multiplication rate of laboratory-adapted *P. falciparum* strains (a 54.4-fold increase in parasitemia over two cycles in untreated blood vs. a 34.5-fold increase in leukocyte-free blood). These observations were made using blood obtained from non-immune donors.

In the present author’s opinion and experience, the presence of leukocytes is a minor technical issue in adapting fresh isolates collected from African patients to in vitro conditions, at least for routine procedures that do not require “pure” parasite DNA for genomic studies. An extra step of leukocyte removal, which may expose the parasites to the risk of bacterial contamination, and additional costs incurred do not seem to offset potential gains from obtaining more rapid adaptation to in vitro cultivation or a higher multiplication rate. As for leukocytes in blood obtained from blood banks in non-endemic countries, leukocyte removal is not necessary for parasite cultivation.

## 6. Serum and Serum Substitutes

Serum remains the principal supplement of culture media in animal cell culture, including malaria cultivation. Classical synthetic media (RPMI 1640, BME and its modifications, MEM, Ham’s F-12, McCoy’s 5A medium) are deficient in some nutrients, such as growth factors, hormones, lipids, and proteins (not amino acids, which are abundant in culture media), which are provided by serum. Depending on the cell line, the following sera have been commonly used: fetal, newborn, or adult bovine serum, human serum, horse serum, and chicken serum. Of these, fetal bovine serum (also referred to as fetal calf serum) is the most widely used in cell culture. Sera from other animals have specialized uses.

In recent years, technical advances have been made to grow animal cells in newly constituted, serum-free, chemically defined media, in modified classical media supplemented with relatively low concentrations (i.e., *v*/*v* < 5%) of serum, or in unmodified classical media supplemented with serum substitutes. The advantages of serum-free media include a completely defined medium, lack of biological variability, absence of blood-borne pathogens, and for malaria cultivation in developing countries, compatibility with any blood type and ease in ordering and transporting a dry medium. Serum-free media are relatively more expensive than classical media. A particular serum-free medium may also be adapted for a limited number of cell lines due to different requirements for serum supplements and basal medium. There is no universal serum-free medium to cultivate all cell lines. In the coming years, serum-free culture will probably become the standard procedure of cell culture or will at least be one of the objectives to be attained for research.

### 6.1. Human Serum

For malaria cultivation, serum or serum replacement is an essential component of the culture medium. In many, but not all laboratories, human serum is used to supplement the culture medium for continuous culture. Trager and Jensen used 15% (*v*/*v*) human serum in the RPMI 1640 medium to establish the first continuous cultures [27]. It has been suggested that 15% (*v*/*v*) human serum may yield better results for initiating culture and for reactivating cryopreserved parasites [16,81]. In their later studies, the concentration of serum was reduced to 10%. The experiences from various laboratories suggest that laboratory-adapted strains do not require more than 10% human serum.

Any major modification of the concentration of human serum in the RPMI 1640 medium leads to suboptimal parasite growth. High serum concentrations (25% and 50%) generally result in suboptimal parasite growth [151]. In a study conducted by Golightly and Greenwood on a synchronized laboratory-adapted *P. falciparum* strain over 24–48 h, increasing serum concentration in the RPMI 1640 medium at a 2.5% increment from 2.5% to 12.5% resulted in a proportional increase in parasitemia, while at higher serum concentrations (15–20%), parasitemia declined [152]. In another study, however, serum concentrations of 10%, 50%, and 80% were shown to result in a similar growth rate of a laboratory-adapted *P. falciparum* strain over a 30–45 h incubation [153]. The long-term effect of high (50–80%) serum concentrations was not studied by these latter investigators. Although malaria parasites survive under a relatively high serum concentration in the human host, the reasons for the suboptimal growth in vitro at 15–50% serum concentration are not known.

As for the minimal serum concentration, Jensen and Trager have suggested that the reduction in serum concentration to 5% results in suboptimal parasite growth [81]. In later studies, it was reported that 5% pooled human serum supports optimal parasite growth of a laboratory-adapted strain, as well as 10% serum, provided that the culture medium is changed daily [154]. Even 2.5% pooled human serum, but not lower serum concentrations, supports parasite growth, albeit at a suboptimal level. A laboratory strain of *P. falciparum* can be adapted to decreasing levels of human serum by progressively replacing the latter with peptone until human serum is completely eliminated [155]. However, the growth rate decreases considerably (8–16%) compared with the control parasites in 10% human serum. Based on the experience of various investigators, the addition of 10% (*v*/*v*) pooled human serum is recommended for routine culture and in vitro drug susceptibility assays.

The nutritional quality of different batches of non-immune human sera to support *P. falciparum* cultures differs considerably. Therefore, depending on the samples, unpooled serum added to RPMI 1640 may result in considerably lower parasite growth (<60%) at 5% serum concentration compared with 10% serum concentration [156]. Human sera obtained from commercial sources may not support parasite growth as much as sera collected in blood banks [27,156]. Since some commercial suppliers accept sending small samples of pooled human serum batches for testing prior to ordering larger quantities of serum, it would be possible to assess the growth of reference laboratory strains of *P. falciparum* in different serum batches before changing serum for cultivation.

Parasite growth varies even with freshly collected individual serum from blood banks. The nutritional deficiencies leading to variations in parasite growth are less apparent when the concentration of individual, unpooled human serum is set at 10% (*v*/*v*). To further optimize the nutritional quality of human serum, it is strongly advised to pool non-immune human serum samples. An adequate pool of human serum is obtained by pooling samples from >10 individual non-immune donors (the more, the better). Some commercial sources of human serum are prepared from a very large number of donors. Even these pooled human serum samples require prior testing to ensure optimal parasite growth. Heat inactivation (56 °C for 30 min) does not improve the nutritional quality of fresh human serum [29,81]. Furthermore, the use of freshly collected human serum stored at +4 °C for more than 2 weeks does not support parasite growth [81]. It is preferable to store aliquots of fresh human serum at –20 °C. Such frozen aliquots can be stored for long periods (i.e., several years) without any loss of their nutritional quality for the parasites.

Serum from the umbilical cord is an alternative source of human serum suitable for cultivation [96,112,113,157,158]. Both laboratory-adapted *P. falciparum* strains and freshly collected clinical isolates grow in a medium supplemented with human umbilical cord serum. Obtaining serum from fresh placenta requires coordination with maternity clinics and hospitals and aseptic techniques. In some populations in malaria-endemic countries, there may be culture-related practices and taboos concerning childbirth, and the placenta may have to be returned to the consenting woman.

For field workers, it is more convenient to procure human sera from local donors. The suitability of locally acquired individual serum samples or pooled samples for in vitro culture needs to be evaluated first. Several early studies have suggested that serum from immune human populations (also serum from *Aotus* monkeys experimentally infected with *P. falciparum*) can inhibit the growth of parasites in vitro, presumably due to the presence of antimalarial antibodies [159,160,161,162,163,164]. In a study on a large number of heat-inactivated and dialyzed sera collected from healthy Sudanese adult volunteers residing in hyperendemic areas, all serum samples inhibited merozoite invasion of a laboratory-adapted reference strain [151,165]. Most sera also produced slower maturation of synchronous parasites as well as morphologically abnormal schizonts (called the “crisis forms”, i.e., a retarded development of asexual intraerythrocytic parasite associated with the host immune response, resulting in the formation of degenerating schizonts containing less than the average number of merozoites per schizont). By contrast, serum samples from healthy Sudanese adults residing in a hypoendemic area did not exhibit any inhibitory effect on parasite growth. In these studies, each test serum was dialyzed to remove about 98% of chloroquine that may eventually have been present in the samples due to self-medication. Thus, any inhibitory effect on parasite growth was attributed to immune factors.

In another study conducted in the field, *P. falciparum* isolates from asymptomatic children in Burkina Faso were initially cultivated for up to 25 h in an RPMI 1640–HEPES–NaHCO_3_ medium supplemented with 10% non-immune human serum, and the schizonts were incubated for an additional 20 h in a medium supplemented with immunoglobulin fractions from enrolled children [166]. Compared with the medium containing non-immune serum, merozoite invasion was inhibited by the immunoglobulins from asymptomatic carriers. The inhibitory effect was more pronounced with heterologous immunoglobulin fractions (i.e., parasite–antibody pairs from different donors) than with autologous immunoglobulins (i.e., parasite–antibody pairs from the same donor). The investigators suggested that their observation may be related to the selection of a parasite population with a low expression of the antigens specifically recognized by the host’s antibodies or infection with parasite strains that had not been encountered before by the host. In another field study, the in vitro growth of a laboratory-adapted *P. falciparum* strain was compared with (i) serum from healthy semi-immune African donors, (ii) acute-phase serum from African malaria-infected patients, and (iii) serum from European and Asian healthy non-immune donors [167]. The parasite growth over three intraerythrocytic cycles was similar with sera from non-immune donors and patients with malaria but was much lower with serum from semi-immune subjects. For initiating the culture of primary isolates and short-term cultivation ex vivo (i.e., for in vitro drug susceptibility assays), the acute-phase autologous serum, i.e., autologous serum matched with the corresponding isolate obtained from the same patient, was shown to be suitable [168]. These observations in the field suggest that sera obtained from populations residing in malaria-endemic areas may be used for in vitro cultivation, at least for short-term cultures. These findings are also in agreement with the World Health Organization (WHO) in vitro macrotest and microtest, which use the patients’ own acute-phase plasma to obtain schizonts for a single intraerythrocytic cycle. However, some studies have suggested that serum from immune and semi-immune humans may be unsuitable for in vitro cultivation [151,165,166]. For long-term *P. falciparum* cultures in malaria-endemic countries, it may be best to avoid the use of sera obtained from local human populations as much as possible and rely on non-immune human serum from donors in malaria-free countries, animal sera, or synthetic serum substitutes. In the following sections, alternative solutions are examined and proposed.

### 6.2. Human Plasma

Serum is plasma minus coagulation factors. Plasma is easier to obtain at blood banks. Whole blood collected in anticoagulant-containing pouches from donors is immediately separated into plasma and cellular components by centrifugation. These fractions are used separately for transfusion, depending on the individual needs of the patients. In contrast, to prepare serum samples, whole blood from donors is collected in pouches without anticoagulant, and coagulated cells are discarded. For blood banks and hospitals, it is more rational to prepare plasma and packed cells, rather than serum, for transfusion.

Human plasma may be used instead of human serum for malaria cultivation [156,169,170]. In the study performed by Hui et al., the growth of laboratory-adapted *P. falciparum* strains in a medium with 10% pooled or unpooled human plasma or 10% human serum was assessed over 5–6 days [171]. There was no difference in parasite growth, and an adaptation period was not necessary for parasites that were previously grown in the medium supplemented with 10% human serum to grow in the medium with 10% human plasma. Read and Hyde compared the rate of parasite growth in media supplemented with untreated pooled human plasma from 2–4-day-old whole blood collected on CPDA, heat-treated plasma (56 °C × 1 h), or serum prepared from plasma [125]. The untreated plasma supported the growth of a reference *P. falciparum* strain slightly better than heat-treated plasma and serum over two schizogonic cycles. Parasitemia was 25–40% higher with fresh, untreated plasma within four weeks of blood donation than plasma stored at –20 °C or –80 °C.

It has already been mentioned in the previous section that sera obtained from semi-immune and immune donors do not seem to support parasite growth [151,165,166]. Likewise, RPMI 1640 supplemented with 10% plasma obtained from malaria-negative Gabonese mothers at the time of delivery or 10% plasma derived from their umbilical cords resulted in a significant decrease (>three-fold decrease) in the growth of a reference strain compared to non-immune plasma [172]. Contrary to the results of these aforementioned studies, heat-inactivated plasma from semi-immune donors residing in endemic areas has been successfully used to initiate a continuous culture of both reference clones and fresh isolates in a study conducted in Nigeria [173]. The growth rate and in vitro drug response of reference clones and newly established strains cultivated in RPMI 1640 supplemented with human serum from non-immune or semi-immune donors were similar. Moreover, the authors claimed to have succeeded in initiating the cultivation of 170 of 250 (68%) fresh isolates and maintaining them in continuous culture by using semi-immune plasma. Although the total number of African donors was not stated in their paper, the authors reported that plasma and erythrocytes from four and three donors, respectively, did not support parasite growth.

If serum is preferred over plasma for malaria cultivation but is not available, serum can be prepared from plasma samples. Clot formation is induced by the addition of CaCl_2_ and thrombin to plasma, and after several hours of incubation at 37 °C, the serum is separated from the clot. Defibrinated human plasma supplemented with hypoxanthine (50 µg/mL), with or without reduced glutathione (600 µg/mL), has been shown to support parasite growth for a short-term duration as much as, or better than human serum [51].

### 6.3. Animal Sera

The original culture method developed by Trager and Jensen required the addition of human serum obtained from non-immune donors to the RPMI 1640 medium (*v*/*v*, 5–15%) [27]. Some laboratories, in particular those in endemic countries, encounter the problem of procuring a constant supply of type AB^+^ human serum from non-immune donors, which in most human populations is not the predominant blood type compared to the other three blood groups. Because of several other disadvantages in obtaining human serum from non-immune donors, including high cost and the requirement to screen for blood-borne pathogens, there have been efforts to search for suitable replacements.

One of the two initial culture systems for *P. falciparum*, that of Haynes et al., was developed by supplementing the culture medium with fetal calf serum [26]. Soon after the development of *P. falciparum* cultivation methods, it has been recognized that the use of human serum, which seemed to yield the best results for parasite growth, is a limiting factor for the wide application of this technique, especially in field laboratories. Several investigators have empirically sought to replace human serum with animal sera, which may be easier to obtain from local abattoirs or commercial sources. In early studies using Trager and Jensen’s cultivation method, it has been shown that some animal sera may support parasite growth [29,156].

A number of studies on the use of animal sera for parasite cultivation followed in the 1980s. Animal sera tested for *P. falciparum* cultivation include the following [26,29,30,81,93,98,99,101,155,156,169,174,175,176,177,178,179,180,181]: fetal calf, calf, adult cow, rabbit, goat, pig, horse, and sheep (Table 2). Human sera can be replaced with animal sera, but the success rate varies with different animal sources. An adaptation period lasting several days to weeks of continuous culture may be required for the parasites to grow in non-human sera [155,174,175]. Once the parasites adapt to animal sera, growth may be sustained for several cycles but may decline over time. In such cases, it may be possible to sustain parasite growth by switching to human sera [176].

Mixed results have been obtained with fetal calf serum, often depending on the source or batches [26,81,98,101,106,155,156,182]. Without supplements (e.g., peptones), gradual adaptation, increased number of sera (15–20%), and/or twice daily medium change, sera from newborn calves and adult cows do not seem to be suitable for parasite cultivation. However, using Ham’s F12 medium supplemented with various human and animal sera (without a neopeptone supplement), Divo and Jensen found that the medium supplemented with 10% pooled adult bovine serum resulted in fair growth (61% of growth supported by 5% pooled human serum in F12 medium) over 96 h compared to F12 supplemented with 5% human serum, and supports continuous culture (tested for 100 days) [93]. Parallel experiments with pooled sera from pigs and goats showed inferior parasite growth over 96 h and failure of continuous culture.

Rabbit serum can replace human serum for long-term cultivation with Trager’s method after the adaptation of the parasites [178]. For short-term (single to three cycles) cultivation and in vitro drug susceptibility assays using laboratory-adapted *P. falciparum* strains, 10–20% rabbit serum may replace 10% human serum to supplement either RPMI 1640 or the combination of RPMI 1640–NCTC–IMDM media (*v*/*v*/*v* 2:1:1) [99,177]. For fresh clinical isolates without prior adaptation to in vitro culture conditions, the addition of 10% rabbit serum to a whole-blood RPMI 1640 mixture (*v*/*v* 1:9) may enhance the ability of the parasites to undergo schizogony during the first intraerythrocytic cycle [183].

In several studies, parasite growth was unsatisfactory with swine, equine, ovine, and caprine sera for short-term and/or long-term cultivation [154,155,156]. In other studies, horse, goat, and sheep sera were found to sustain parasite growth for several weeks, with or without an adaptation period [29,99,169,175,181]. However, in one study using fresh clinical isolates cultivated for a single intraerythrocytic cycle, one batch of goat serum proved to be a suitable serum substitute, while horse serum did not support parasite growth [103]. In contrast, in another study using the reference *P. falciparum* clone 3D7, RPMI 1640–10% goat serum was unsuitable to sustain parasite growth for a short-term period (three cycles), while RPMI 1640–10% horse serum supported the parasite growth [99]. These contradictory results may have been due to batch-to-batch differences in the nutritional quality of animal sera. Another possible explanation may lie in anti-human erythrocyte antibodies in animal sera [184]. The latter can be removed by citration and adsorption with washed human erythrocytes, as described by these authors. Calf serum and horse serum treated with citrate and adsorbed with uninfected human erythrocytes before use for *P. falciparum* cultivation were reported to support parasite growth to a similar extent as human serum. This method has not been widely adopted by malaria researchers to date.

For short-term cultivation (one or two cycles), monkey serum (owl monkey (*Aotus trivirgatus*) or squirrel monkey (*Saimiri sciureus*)) has been shown to support the growth of laboratory-adapted *P. falciparum* strains to some extent, or as well as human serum, without any adaptation period [117,153,159,163]. However, for obvious reasons related to animal protection and rights and the availability of other serum sources, the use of monkey serum for routine cultivation of *P. falciparum* is not recommended.

In the experiments conducted by Sax and Rieckmann, a two-to-three-week adaptation period was required for three of four human serum-adapted *P. falciparum* strains to grow in a rabbit serum-supplemented RPMI 1640 medium at a similar rate as a human serum-supplemented RPMI 1640 medium [178]. One *P. falciparum* strain did not require any transition period for adaptation to rabbit serum. The RPMI 1640 medium containing 5 or 10% rabbit serum yielded satisfactory parasite growth, while lower (2.5%) or higher (15%) concentrations of rabbit serum were associated with lower parasite growth. For investigators planning to use rabbit serum instead of human serum, it is advisable to perform preliminary tests on different rabbit sera batches and use pooled serum samples.

Several, but not all, investigators have found that fetal calf serum is a suitable substitute for human serum in *P. falciparum* culture. Like human serum, batch-to-batch variation in its ability to support in vitro parasite cultivation exists. In the experience of Trager and Jensen, none of the three different lots of commercially available fetal calf serum supported parasite growth as much as human serum [115]. Moreover, the growth rates in fetal calf serum-supplemented RPMI 1640 diminished with time. This latter phenomenon was also reported by another research team [101]. Their observation is not in total agreement with the experience of some other researchers, who have obtained satisfactory long-term cultivation with fetal or newborn calf serum [26,30,98,155]. Another study has also demonstrated that a large majority of fresh clinical isolates undergo schizogony during the first intraerythrocytic cycle in RPMI 1640 supplemented with 10% fetal calf serum [106,182].

It may be concluded that among animal sera, fetal calf serum is probably the best substitute for human serum for the cultivation of intraerythrocytic stages of *P. falciparum*. All human blood types are compatible with fetal calf serum. Moreover, large quantities of fetal calf serum are available at a reasonable cost through reliable commercial sources that usually provide proof of mycoplasma-negative samples. As for human serum, batch-to-batch variation in the capacity of fetal calf serum to support in vitro propagation of *P. falciparum* exists. A preliminary evaluation of each batch of fetal calf serum is required to ensure optimal culture conditions.

### 6.4. Serum-Free Culture

Human serum can be replaced with serum substitutes, some of which are commercially available. There are obvious advantages of using serum replacements: limited batch-to-batch variation, which is a prerequisite for a standardized protocol; compatibility with any human blood type; production at industrial scale and availability through commercial sources, usually at lower costs than non-immune human serum; ease in handling and transport; limited risks of blood-borne pathogens, such as human immunodeficiency virus and hepatitis virus. Serum replacements with which in vitro cultivation of *P. falciparum* has been achieved are listed in Table 3 [54,99,101,122,155,156,167,174,185,186,187,188,189,190,191,192,193,194,195,196,197,198,199].

One of the earliest attempts to replace human plasma with fatty-acid-free bovine or human albumin or stearic acid suggested the suitability of bovine albumin, and to a lesser extent, human albumin and stearic acid, to support the in vitro development of *P. falciparum* and/or *P. knowlesi* for a single cycle [185,186,201]. These experiments were conducted with *P. knowlesi* maintained in *Macaca mulatta* or *P. falciparum* maintained in *Aotus trivirgatus* and were cultivated in vitro in the now outdated Harvard medium supplemented with plasma or serum substitutes. Jensen reported that the use of fatty-acid-free bovine or human serum albumin as a replacement for human serum in RPMI 1640 did not result in satisfactory parasite growth with the candle jar technique [156]. Another serum substitute, composed of Bacto-peptone, Yeastolate, lactalbumin hydrolysate, bovine insulin, and polyvinylpyrrolidone, resulted in poor parasite growth with the candle jar method.

*Plasmodium falciparum* strains were successfully maintained for more than one month in a serum-free medium with unsaturated C-18 fatty acids added to the RPMI–HEPES–NaHCO_3_ medium containing 6 g/L bovine albumin and 10 mg/L adenosine [188]. Among the fatty acids tested, *cis*-vaccenic, oleic, and linoleic acids supported parasite growth at the concentration of 10 µM (approximately 3 mg/L). Lower (0.1–5 µM) or higher (up to 100 µM) concentrations resulted in either suboptimal growth or no growth. The growth rate in a medium supplemented with one of the three fatty acids was about half of the control cultures maintained in the medium supplemented with 10% human plasma. Various combinations of unsaturated C-18 fatty acids added to the medium did not improve the growth rate. It was confirmed in the study that, as in previous studies, bovine albumin, alone or in combination with stearic acid, did not support parasite growth beyond a few cycles. Furthermore, in their earlier work (unpublished data cited in [188]), saturated fatty acids from C-14 to C-24 did not support the in vitro growth for a single intraerythrocytic cycle.

An established strain initially adapted to the RPMI 1640 medium supplemented with 10% human serum can be gradually adapted to grow in a serum-free RPMI 1640 medium containing peptone additives (Neopeptone^®^ or Proteose-Peptone^®^ no. 3; 0.15% *w*/*v* final concentration) over a one-month period [155]. However, the parasite growth rate was much lower than in human serum-supplemented RPMI 1640. After adaptation, when 5–10% fetal calf serum was added to either Neopeptone^®^ or Proteose-Peptone^®^ no. 3, long-term cultivation with parasite growth comparable to, or better than, that observed with 10% human serum was possible. That study has also shown that human serum fractions II (gamma globulins), IV (α globulins), and V (albumin) used as a replacement for human serum do not improve parasite growth. Peptones can be replaced by 50 µM hypoxanthine [96].

One of the key steps necessary to attain the serum-free culture of *P. falciparum* is to identify human serum component(s) required to satisfy the parasites’ nutritional needs. In short-term experiments with a laboratory-adapted strain grown in a “basic” RPMI 1640 medium (supplemented with fatty acid-free bovine serum albumin (5 mg/mL) and 10% dialyzable human serum factors), parasites did not grow even for 48 h in the absence of nondialyzable macromolecules [187]. The addition of seric lipoproteins (low-density lipoproteins (LDLs) and high-density lipoproteins (HDLs)) to the medium supported parasite growth, but not to the extent of the control medium with 10% human serum. The continuous culture was not performed with the lipoprotein-supplemented “basic” medium in that study. Further studies showed that the human HDL fraction is the major lipid source for *P. falciparum* during its intraerythrocytic cycle [189,202]. At the concentration of 0.25–0.5 mg/mL HDL, this lipoprotein fraction was shown to support parasite growth for two complete cycles in the RPMI 1640–HEPES–NaHCO_3_ medium supplemented with 50 µg/mL hypoxanthine and 600 µg/mL reduced glutathione (2% starting parasitemia, 4% hematocrit, 6% O_2_–3% CO_2_ gas mixture) to a similar level as the culture medium containing 5% human serum. The other two lipoprotein fractions, LDLs and very-low-density lipoproteins (VLDLs), did not support parasite growth and induced hemolysis. Long-term cultivation was not performed in that study. In a more recent study [203], it was confirmed that human serum HDLs at a concentration of 0.75 mg/mL, but not LDLs, can replace whole human serum for short-term (72 h) cultivation. At a higher concentration of 3 mg/mL, HDL was toxic.

A chemically defined fraction of adult bovine serum, commercialized as “Daigo’s GF21” (Wako Pure Chemical Industries Ltd., Osaka, Japan), has been used as a serum substitute for in vitro cultivation of various cell lines. This product has been reported to be a promising serum substitute for malaria cultivation [101]. It consists of a 55–70% ammonium sulfate fraction of adult bovine serum supplemented with insulin, transferrin, ethanolamine, and sodium selenite. For optimal cell growth, Daigo’s GF21 is diluted to 10% (*v*/*v*) in an appropriate culture medium (RPMI 1640 and others, depending on the cell culture), which contains an equivalent of 3 g of protein/L. For malaria cultivation, RPMI 1640 supplemented with Daigo’s GF21 results in suboptimal growth. The best result is obtained with Daigo’s T basal medium (a modified mixture of IMDM and the F-12 medium) supplemented with 10% (*v*/*v*) Daigo’s GF21 [101].

The same manufacturer also produces the GIT medium, a ready-to-use culture medium composed of an equal volume of IMDM and Ham’s F-12 medium, insulin, transferrin, ethanolamine, sodium selenite, and 10% (*v*/*v*) GF21. The composition of the GIT medium is almost identical to Daigo’s T basal medium supplemented with 10% (*v*/*v*) Daigo’s GF21. An addition of fetal bovine serum (0.1–3%) may further enhance the in vitro growth of certain cell lines. In short-term cultures of laboratory-adapted *P. falciparum* strains and field isolates, parasite growth was poor in RPMI 1640 supplemented with GF21. In contrast, parasite growth was similar in the GIT medium and RPMI 1640–10% human serum for laboratory strains and better in the GIT medium than in RPMI 1640–10% human serum for fresh clinical isolates [101,103]. Furthermore, the Japanese investigators reported successful continuous cultivation of two *P. falciparum* strains in an unsupplemented GIT medium for more than a year [101]. These laboratory strains readily adapted to the GIT medium. Among 12 lots of the GIT medium tested for malaria cultivation, there was no batch-to-batch variation in supporting parasite growth. This medium has also been used by another Japanese research team to maintain an additional reference clone of *P. falciparum* for long-term cultivation [204].

Nutridoma-SR (Boehringer Mannheim, Mannheim, Germany; now Roche Diagnostics) is a biochemically defined supplement designed to replace fetal calf serum for cell cultures. It is composed of human proteins, insulin, transferrin, cholesterol, and organic and inorganic compounds. In the initial study designed to induce gametocyte production in a serum-free medium, Lingnau et al. observed that a laboratory strain of *P. falciparum* grows readily without any adaptation period in the serum-free RPMI 1640–HEPES–NaHCO_3_ medium supplemented with 50 mg/L hypoxanthine and Nutridoma-SR (10% hematocrit, 3% O_2_–5% CO_2_) [193]. Parasite growth in the Nutridoma-SR-supplemented RPMI 1640 medium was concentration-dependent within the range of 0.5% and 4% Nutridoma-SR. Using 4% Nutridoma-SR, the asexual parasites grew to a similar extent in RPMI 1640 supplemented with 10% human serum during the 14-day cultivation period with daily medium change (fresh erythrocytes were not added in this study to enhance gametocyte production). Lower concentrations of Nutridoma-SR resulted in suboptimal growth. In the follow-up study designed to evaluate the suitability of Nutridoma-SR as a serum substitute for asexual parasite propagation, several *P. falciparum* strains grew readily in RPMI 1640 supplemented with 50 mg/L hypoxanthine and 4% Nutridoma-SR (8–10% hematocrit, 3% O_2_–5% CO_2_) [190]. Compared with the growth in the standard RPMI 1640–HEPES–NaHCO_3_ medium supplemented with 10% human serum, the growth in the Nutridoma-supplemented RPMI 1640 was similar or better in three of four parasite strains over two to three weeks of continuous culture. One strain developed in both media, but the growth rate was much higher in RPMI 1640 supplemented with human serum.

These findings were not fully confirmed by another group. Under similar experimental conditions (but at lower hematocrit (2%) and 5% O_2_–5% CO_2_ gas mixture), Flores et al. were unable to cultivate three different laboratory-adapted *P. falciparum* strains beyond a few intraerythrocytic cycles using the RPMI 1640–HEPES–NaHCO_3_ medium supplemented with 0.2% d-glucose and either 4% Nutridoma-SR or Albumax^®^ I [191]. A sustained in vitro growth was obtained by combining Nutridoma-SR and Albumax^®^ I with an optimal growth rate observed with 1% (*v*/*v*) Nutridoma/0.25% (*w*/*v*) or 0.5% Albumax^®^ I. These combinations of serum supplements allowed a continuous culture for more than one month. No further studies seem to have been performed with Nutridoma as a serum substitute for malaria cultivation.

Some other serum substitutes have been assessed for their suitability to support *P. falciparum* cultures. Ultroser^®^ (Invitrogen Gibco Life Technologies) is a serum replacement containing binding proteins, fatty acids, phospholipids, adhesion factors, hormones, vitamins, and growth factors [200]. The manufacturer recommends 2–4% Ultroser^®^ for serum-free cultures of mammalian cells. In a study designed to assess parasite growth for two complete cycles, Mohapatra and Hommel found that 1–4% Ultroser^®^ G in RPMI 1640, as well as 1–2% Ultroser^®^ combined with 1–5% human serum, did not support parasite growth [192]. The addition of low Ultroser^®^ concentrations ≤0.5% together with a low concentration of human serum (≤2%) led to satisfactory parasite growth for 4 days. In another study, RPMI 1640 supplemented with 0.5–4% Ultroser^®^ G also did not support asexual parasite growth for the first three days [193]. The use of 0.5–1% Ultroser^®^ G, but not 2–4% Ultroser^®^ G, seemed to support the growth of asexual parasites when cultivation was pursued beyond 5 days. Ultroser^®^ SF, which is identical to Ultroser^®^ G except for the omission of steroids, also supported parasite growth to a similar extent. Ultroser^®^ HY did not support parasite growth [106,193]. It is necessary to reemphasize that the experiments conducted by Lingnau et al. [193] were designed to induce gametocytogenesis and cultivate gametocytes. The study was, therefore, not designed for the optimal growth of asexual parasites (i.e., fresh uninfected erythrocytes were not added during the 15-day cultivation period). Further attempts to replace human or animal serum with Ultroser^®^ serum substitute for the cultivation of asexual *P. falciparum* parasites do not seem to have been made.

Ofulla et al. reported that the growth rate of a laboratory-adapted strain cultivated in the RPMI 1640–HEPES–NaHCO_3_ medium supplemented with 5 g/L of fatty acid-free bovine albumin with daily medium change for five days (initial parasitemia 0.1%, hematocrit 5%) attained almost 90% of that with 10% human serum [54]. Long-term cultures were not performed with this medium preparation. Growth rates were much lower or nil (<60%) at higher (10–20 g/L) or lower (0.2–4 g/L) concentrations of fatty acid-free bovine albumin. The use of a 5 g/L Cohn fraction V of bovine albumin, which contains fatty acids, improved the short-term parasite growth compared with fatty-acid-free bovine albumin. An even better growth of the parasites that is comparable with that in 10% human serum was observed with the medium supplemented with a 5 g/L Cohn fraction V of bovine albumin and a lipid–cholesterol-rich mixture. The latter mixture, which contains fatty acids, cholesterol, lipoproteins, and phospholipids derived from bovine serum, supports parasite growth better than 3 mg/L *cis*-vaccenic, oleic, and linoleic acids, individually or in combination, which were previously tested by Willet and Canfield [188]. The RPMI 1640–HEPES–NaHCO_3_ medium supplemented with 2 g/L extra d-glucose, 60 mg/L cold hypoxanthine, a 5 g/L Cohn fraction V of bovine albumin, and a 10 mL/L lipid–cholesterol-rich mixture was successfully used for long-term cultivation of several *P. falciparum* strains. The findings of Ofulla et al. paved the way for the use of a similar supplement derived from bovine serum, Albumax^®^, which is available through a commercial source (Invitrogen Gibco Life Technologies, Paisley, United Kingdom) [54].

Most of the serum replacements mentioned in this section have been evaluated in individual laboratories. The exception is Albumax^®^ (available as Albumax^®^ I and Albumax^®^ II; the difference is the percentage of residual immunoglobulins), which has gained popularity among malaria researchers, both for continuous culture and in vitro drug susceptibility assays. The use of Albumax^®^ as a replacement for human serum eliminates the potential problem of ABO compatibility between erythrocytes and serum and is cheaper than type AB human serum. It is readily available through an international supplier, may be transported to the field at ambient temperature, and is stable. Some of its disadvantages include the unknown exact composition, possible batch-to-batch differences in supporting parasite growth observed by some investigators, and differences in drug–protein binding properties between bovine albumin (and other proteins) and human serum, leading to widely different in vitro responses for some antimalarial drugs. Albumax^®^ is usually diluted to a final concentration (*w*/*v*) of 0.5% in the RPMI 1640 medium. The concentration of Albumax^®^ is based on the observation that human serum contains approximately 50 g/L albumin, which is diluted to 5 g/L (=0.5%) in the RPMI medium with 10% serum. The RPMI 1640–Albumax^®^ liquid mixture can be stored at +4 °C for up to 2 months [194].

Several research groups have demonstrated that various laboratory-adapted *P. falciparum* strains initially adapted to the RPMI 1640/human serum medium can be adapted with ease to the RPMI 1640/Albumax^®^ medium. Although one of the first attempts to adapt several strains to RPMI 1640/0.5% Albumax^®^ I was a failure without additional supplements (i.e., Nutridoma-SR) [191], in subsequent experimental studies performed by other research groups, it was reported that several *P. falciparum* strains, including reference clones (HB3, 3D7, W2, Dd2), were maintained in a continuous culture in the RPMI 1640/0.5% Albumax^®^ I medium, but technical details were not provided [195,196]. The growth of more than 10 different *P. falciparum* strains in RPMI 1640/0.5% Albumax was reported to be comparable to RPMI 1640/10% human serum, especially when the former medium was supplemented with hypoxanthine [194]. For short-term cultivation (i.e., up to 7 days; starting parasitemia, 0.5%; hematocrit 6%; replacement of culture media every 48 h) of a laboratory-adapted strain (3D7 clone), RPMI 1640 supplemented with Albumax^®^ II at low concentrations (0.01%, 0.05%, and 0.1%) supported parasite growth to a similar extent as RPMI 1640/0.5% Albumax^®^ II [99]. Another laboratory-adapted strain (D6/Sierra Leone) was easily adapted to grow continuously for more than two months in the RPMI 1640/0.5% Albumax^®^ I medium and yielded higher parasitemia than the control parasites grown in RPMI 1640/10% human serum [197]. Fresh isolates of *P. falciparum* have also been adapted to grow in RPMI 1640/0.15% Albumax^®^ (one isolate “BINH/Kenya” was maintained for more than 2 years), RPMI 1640/2.5% Albumax^®^ I/0.5 g/L hypoxanthine (5 of 38 Malian isolates for 3–4 weeks), or RPMI 1640/0.5% Albumax^®^/0.05% hypoxanthine (21 of 33 Indian isolates for up to 35 days; 10 of 12 cryopreserved Indian isolates for up to 45 days) in a continuous culture [167,198,199].

RPMI 1640 supplemented with 0.5% Albumax^®^ has also been used successfully to perform in vitro (i.e., with laboratory-adapted strains) or ex vivo (i.e., with fresh clinical isolates without prior adaptation to in vitro culture) drug susceptibility assays (i.e., 48–72 h short-term cultivation) [106,168,197,205,206,207,208]. Some authors have reported that IC_50_ values for different antimalarial drugs obtained with RPMI 1640/human serum and RPMI 1640/Albumax^®^ media may be significantly different, probably due to differences in drug binding properties of albumin present in human serum and Albumax^®^ [106,168,205]. The aforementioned studies suggest that most, if not all, strains already adapted to continuous culture in RPMI 1640/human serum, as well as fresh isolates that can be adapted to in vitro culture conditions with RPMI 1640/human serum and can probably be also adapted to the RPMI 1640/Albumax^®^ medium.

Human serum contains 40–50 g/L of albumin [197], and compared to Albumax^®^ (lipid-enriched bovine serum albumin), has more phospholipids and cholesterol, but less fatty acid [209]. The composition of human serum is also much more complex (it contains a variety of amino acids, proteins, lipids, carbohydrates, and electrolytes) and is probably more nutritive, but there is a wide interindividual variation. The combined supplement of human serum and Albumax^®^ to RPMI 1640 increases the protein content to support parasite growth, especially freshly isolated parasites that are adapted to in vitro cultivation during the initial intraerythrocytic cycles. For continuous cultivation under optimal conditions and/or for rapid adaptation to serum-free conditions, some investigators recommend the addition of hypoxanthine (40–200 µM) [167,194]. Binh et al. observed that, compared with the parasite growth in Albumax^®^-supplemented RPMI 1640, a two-to-five-fold greater growth rate is obtained when 10% non-immune human serum or 10% autologous serum from patients with acute malaria is added (i.e., 10% human serum + 1.5% Albumax^®^) [167]. For short-term cultivation (72 h), a combined supplement of 10–15% human serum, 0.5% Albumax^®^ II, and hypoxanthine supported parasite growth twice as much as Albumax^®^ II alone, but much less than 15% human serum [210]. Another research group recently reported that a 1:1 mixture of human serum and Albumax^®^ II supports parasite growth (based on the multiplication factor) better than, or equivalent to, either 10% human serum alone or Albumax^®^ II alone supplemented to the standard culture medium (RPMI 1640, 25 mM HEPES, 25 mM NaHCO_3_) [211]. The mixed supplement (Albumax^®^ + human serum) may not necessarily be beneficial. Depending on the parasite strain and how it was adapted to different culture media, different results may be obtained. For example, Singh et al. observed that once D6/Sierre Leone clone was adapted to a culture medium with only 0.5% Albumax after 2 months of continuous cultivation, parasite growth was better (i.e., with two-fold higher parasitemia) with RPMI 1640 supplemented with 0.5% Albumax^®^ I than RPMI 1640 supplemented with 10% human serum [197].

The use of serum-free culture media in experiments has raised some concerns about the possible altered biology of malaria parasites. In one study, transcriptome analysis using microarrays in a reference strain grown in RPMI 1640/human serum or RPMI 1640/Albumax^®^ did not reveal any major difference in the gene expression profile [197]. Those authors found 43 genes with increased expression in parasites cultivated with Albumax^®^. This result was not confirmed in another study in which transcriptome analysis was performed by RNA sequencing [212]. In the latter study, cultivation in RPMI 1640 supplemented with Albumax^®^ resulted in an increased expression of more than 500 genes and parasites with fewer knobs and decreased cytoadherence. These results tend to be supported by another series of experiments carried out by Ribacke et al., who recommended that, based on their findings, *P. falciparum* parasites should be grown in RPMI 1640/10% human serum rather than RPMI 1640/0.5% Albumax^®^ for optimal conditions in experiments on cytoadherence and rosetting [213]. Other authors have reported that the replacement of human serum with Albumax^®^ to cultivate the reference clone 3D7 results in a decreased rate of erythrocyte invasion, increased duration of the life cycle (40 h with 5% human serum + 0.25% Albumax^®^ II versus 45 h with 0.5% Albumax^®^ II alone), and altered drug response [214,215].

The genetic basis of alteration in parasite biology in different culture conditions was recently explored. Several gene candidates (e.g., aspartate transaminase, erythrocyte-binding antigen-140, cysteine protease autophagy-related 4) in three loci have been associated with differential growth rates in two laboratory-adapted *P. falciparum* clones in the standard RPMI 1640 medium supplemented with either 10% human serum or 0.5% Albumax^®^ II [209]. These observations suggest that biological fitness, defined as the capacity to proliferate competitively (i.e., the capacity to outgrow the other parasites when placed in the presence of other parasite populations or strains), may be at least partly dependent on culture conditions, especially serum supplements or serum substitutes. These recent experimental studies may be the first step to pave the way to identify the genes associated with biological fitness and nutritional requirements of the parasites maintained under different culture conditions.

The fractionation of serum components and the reconstitution of lipid-free serum albumin and various combinations of fatty acids have led to the discovery that the key components provided by human or animal serum and Albumax^®^ may be serum albumin, oleic acid, and palmitic acid [216]. These three components, at a concentration of 30 µM each, supported the growth of a laboratory-adapted strain for over one month, at a growth rate that is 20% less than the control culture maintained with 10% human serum. The optimal ratio of serum albumin and two fatty acids is still undetermined. These essential fatty acids are metabolized into lipids (phosphatidylcholine, phosphatidylethanolamine, diacylglycerol, triacylglycerol) that are major constituents of membranes in the parasite. In a follow-up study, six combinations of human serum-derived fatty acids that can replace whole human serum for a continuous culture for over six months of a reference *P. falciparum* strain were identified [217]. These findings help to understand which components of the human serum are required for the development of *P. falciparum* parasite and, hopefully, will be a step forward toward the development of a chemically defined, serum-free medium for optimal malaria cultivation of various parasite strains and fresh clinical isolates.

## 7. Hypoxanthine

Hypoxanthine, a purine base precursor of adenine and guanine that readily enters intraerythrocytic malaria parasites, has been identified as an important serum component necessary to enhance in vitro growth [218,219,220,221]. Although other purines can replace hypoxanthine, the RPMI 1640 medium does not contain purines. The addition of human serum and human erythrocytes was thus the only source of hypoxanthine during the early phase of the cultivation method developed by Trager and Jensen [27]. In the alternative cultivation method developed by Haynes et al. [26], the medium that they used, medium 199 with Earle’s salts, contains 0.354 mg/L of hypoxanthine.

Human serum or plasma dialyzed through membrane pore size corresponding to low molecular mass <6000–8000 (which eliminates purines, pyrimidines, peptides, and mineral salts) results in poor parasite growth when it is used to supplement RPMI 1640 [93,156]. By contrast, media containing hypoxanthine, such as Ham’s F12 (4.77 mg/L), support parasite growth even when they are supplemented with exhaustively dialyzed human serum. By adding different components that are present in Ham’s F12, but not in RPMI 1640, Divo and Jensen demonstrated that the key dialyzable low molecular weight component that is not supplied by RPMI 1640 but is necessary for enhanced parasite growth is hypoxanthine [93]. These findings were confirmed in other experiments [222]. The optimal concentration of hypoxanthine to support parasite growth in RPMI 1640 supplemented with pooled adult bovine serum (which contains 6 × 10^−7^ M [0.6 µM] of hypoxanthine) was 3–12 × 10^−5^ M (30–120 µM) [93]. In the experiments conducted by Asahi et al. [222], the optimal hypoxanthine concentration was estimated to lie between 15 and 120 µM. In other experiments, the lowest concentration of exogenous hypoxanthine required to complete one intraerythrocytic cycle (in RPMI 1640 with presumably 10% human serum) was determined to be 2 µM; optimal growth of 3D7 clone required ≥5 µM of hypoxanthine [223]. Other purines, such as inosine, adenosine, and, to a lesser extent, adenine, also support parasite growth in RPMI 1640 supplemented with 10% pooled adult bovine serum. In comparison, the concentration of hypoxanthine in RPMI 1640 supplemented with human serum is 1.5–2.0 × 10^−5^ M (15–20 µM) [224]. The concentrations of hypoxanthine in human plasma and erythrocytes have been estimated to be 1840 ± 1320 nM (1.84 ± 1.32 µM) and 11,100 ± 7600 nM (11.1 ± 7.6 µM), respectively [225]. Human erythrocytes contain, on average, a six-fold (up to 10-to-15-fold) higher concentration of hypoxanthine compared to the plasma level.

The effect of hypoxanthine added to RPMI–TES–NaHCO_3_ with high glucose concentration (4 g/L) on the growth of laboratory-adapted strains in suspension culture (10% erythrocyte, 0.8–1.5% starting parasitemia) was studied over four days [51]. The growth of a reference *P. falciparum* clone increased with increasing hypoxanthine concentration between 0.5 and 5 µg/mL, remained constant at concentrations between 5 and 50 µg/mL, and decreased at higher hypoxanthine concentrations of 50–100 µg/mL. However, the growth rates of three uncloned Thai isolates were not affected by the addition of hypoxanthine. Another research group studied the effect of low hypoxanthine (“hypoxanthine-deprived medium” with 0.5 µM hypoxanthine in RPMI 1640/0.5% Albumax^®^ II versus 90 µM hypoxanthine in the control parasites) on the growth of a laboratory-adapted strain (NF54^attB^) over three intraerythrocytic cycles [226]. During the first 24 h in a hypoxanthine-deprived medium, parasitemia and morphology under an optical microscope remained comparable to the control parasites. Cultivation for 40 h in a hypoxanthine-deprived medium resulted in a decrease in parasitemia by about 50% (when assessed at 72 h), delayed schizogony, and abnormal morphology (presence of vacuole-like structures). The parasites cultivated in a hypoxanthine-deprived medium for 40–72 h, but not more than 72 h, were able to complete the second intraerythrocytic cycle if placed in the control RPMI 1640 with 90 µM of hypoxanthine.

When the standard RPMI 1640–HEPES–NaHCO_3_–10% human serum (or plasma) mixture is supplemented with l-glutamine (5 mM) and hypoxanthine (50 mg/L), it is possible to maintain the continuous culture of a laboratory-adapted strain for up to three days without medium change, provided that the initial parasitemia (≤0.5%) and hematocrit (2.5%) are sufficiently low [227]. Furthermore, cultures in hypoxanthine-supplemented (0.37 mM) RPMI 1640/10% human plasma at 2.5% hematocrit and 0.3% initial parasitemia without medium change for 72 h grow to a similar extent as the control cultures (without hypoxanthine supplement) with daily medium replacement [170]. The addition of hypoxanthine may not be indispensable for cultures left for several days without daily medium replacement, provided that 10% human plasma or serum is used. In one study, cultures of a laboratory-adapted strain (5% hematocrit and 0.5% initial parasitemia) grew without medium change over the weekend (i.e., three days) to the same extent as the parallel cultures with once daily medium change using the standard medium supplemented with l-glutamine, 2 g/L extra glucose (final concentration, 4 g/L), and 10% human plasma, even without hypoxanthine supplement [125].

Based on the observations of several investigators, it may be stated that, in general, the addition of hypoxanthine leads to improved growth rates [51,54,93,101,194]. Despite these observations, hypoxanthine supplementation does not seem to be an absolute requirement for a successful culture, as short-term and long-term cultivation is possible without additional hypoxanthine as long as RPMI 1640 is supplemented with 10% human serum or plasma. It may be hypothesized that purine stores in the host erythrocyte and serum (or plasma) may provide a sufficient source of hypoxanthine. Nonetheless, for the routine cultivation of *P. falciparum* in RPMI 1640/10% human plasma or serum, an addition of hypoxanthine is beneficial to maintain a high growth rate. The situation is different if RPMI 1640 is supplemented with Albumax^®^. In this latter case, it is highly recommended to add hypoxanthine to the culture medium.

It should be noted that the presence of a “cold” hypoxanthine supplement in the culture medium may interfere with the incorporation of [^3^H]hypoxanthine in radioisotope-based drug susceptibility assays [49,50]. The usual practice is to provide a wash-out period of at least one complete intraerythrocytic cycle during which the parasites are cultivated in RPMI 1640 without hypoxanthine supplement, or the infected erythrocytes are thoroughly washed in the hypoxanthine-free medium before performing isotopic drug susceptibility assays.

## 8. Feeder Cells

Feeder cells refer to a monolayer of adherent animal cells that secrete proteins and other metabolites that supplement the culture medium to enhance the growth of a co-cultivated cell line. Feeder cells are first grown to confluency and irradiated to halt their proliferation. Another cell line, which is usually difficult to cultivate without extensive supplementation of the medium, is added to the feeder layer for co-cultivation.

This specialized technique has been applied to malaria cultivation. Early studies performed during the 1980s showed that when laboratory-adapted strains or fresh clinical isolates are cultivated on monolayers of murine hepatocytes, fibroblasts, or macrophages, or co-cultivated with human peripheral blood mononuclear cells, it was observed that parasites grow at a higher rate, attain higher parasitemia, and adapt better to in vitro culture conditions [157,158,228,229,230]. Instead of co-cultivation, another approach is to cultivate mouse hepatocytes separately and add the supernatant (*v*/*v* 5–15%) to *P. falciparum* cultures (RPMI 1640/10% human serum) [231]. The authors reported that the growth rate over a single intraerythrocytic cycle increased significantly (a mean of 1.4–1.5-fold) with the addition of 10–15% (*v*/*v*) supernatant in both laboratory-adapted strains and freshly collected isolates. More recently, the co-cultivation method of B lymphocytes and *P. falciparum* has been further refined [232]. The authors reported that activated B lymphocytes and laboratory-adapted *P. falciparum* strains can be co-cultured in direct contact in IMDM and, to a lesser extent, in RPMI 1640 and DMEM, supplemented with 5% human serum and 5% fetal bovine serum for up to 10 days. Co-culture enhanced cell growth mutually, with higher parasitemia and more rapid growth of *P. falciparum* and enhanced B-cell proliferation.

The underlying mechanism leading to improved growth conditions in co-cultures is not well understood. Feeder cells may provide additional nutrients that are either absent or insufficient in the RPMI–10% serum–erythrocyte mixture. These ”sophisticated” techniques are not commonly used in most malaria research laboratories. For routine cultures, the use of feeder cells is not required.

## 9. Static versus Suspension Culture

In malaria blood cell cultivation, the term “static culture” means malaria-infected erythrocytes cultivated without agitation. A thin layer of erythrocytes is deposited on the flat-bottomed surface of the culture vessel, plates, flasks, or Petri dish, without any form of adhesion to the substrate. The term “suspension culture” refers to a constant or intermittent agitation of malaria-infected erythrocytes so that the cells remain suspended in the culture medium.

The rocker-dilution method, which involves a constant agitation of infected erythrocytes suspended in the culture medium, was used in early experiments on short-term cultures. Based on the observation that, in vivo, *P. falciparum*-infected erythrocytes remain attached to the endothelium of microvessels in various organs only during the more mature growth stages of the developing schizont, Trager and Jensen reasoned that alternative methods are more appropriate for the long-term cultivation of *P. falciparum* [27]. Two methods have been introduced: the more cumbersome, continuous flow method using a special flow-vial which injects a slow flow of medium over a settled layer of erythrocytes and the more practical, Petri dish-candle jar method, which is stationary. The current standard procedure involves a settled layer of erythrocytes under static conditions during the entire incubation period. The only moment when the erythrocytes are agitated into suspension is during the medium change in Petri dishes or culture flasks, once the spent medium is aspirated and the fresh medium is added.

In the human host, erythrocytes infected by *P. falciparum* rings are in constant circulation in the bloodstream (i.e., in suspension), whereas erythrocytes infected by mature trophozoites and schizonts are stationary due to cytoadherence in deep organs. In vivo, it may be hypothesized that a slow, constant flow of blood over stationary schizont-infected erythrocytes may be more favorable for the dispersion of merozoites devoid of motile organelles to initiate a new intraerythrocytic cycle in a higher number of erythrocytes. In contrast, merozoites released in a completely stationary environment, such as Petri dishes, tend to agglomerate and invade the same uninfected erythrocyte; multiple infections indicate that many merozoites penetrate close to the point of schizont rupture. Erythrocytes infected with two or more rings and trophozoites are more commonly observed in the stationary culture compared with the suspension culture [233].

During early attempts to develop malaria cultivation methods, most cultures had been performed in Erlenmeyer flasks in an atmosphere of 5% CO_2_ in the air and placed on a rocking table in an incubator [16]. The rocker flask method does not yield satisfactory parasite growth. In the method developed by Trager and Jensen [27], a stationary, settled thin layer of infected erythrocytes is cultivated on the flat-bottomed surface of either a flow vial or a Petri dish. In later works performed in the late-1970s and 1980s, several investigators compared static and suspension cultures of established *P. falciparum* strains and concluded that cultures on an orbital shaking device or flask shaker led to a higher yield of parasites [29,51,53,109,233,234]. With suspension cultures at 10% erythrocyte suspension (*v*/*v*), the yield may be a two-fold increase over static cultures during short-term (two to three intraerythrocytic cycles) and long-term (one month) cultures [51,233]. In suspension cultures maintained at a lower erythrocyte suspension (*v*/*v*, 1%) and containing high glucose concentration (extra 2 g/L in addition to 2 g/L in RPMI 1640; 40 mM TES), higher parasitemia can be obtained compared to static cultures, without changing the medium for 3–4 days [53]. These early studies support the observation that the suspension culture generally results in a higher yield of parasites. In one study based on the comparison of the in vitro growth of three parasite strains maintained in static and suspension cultures, the benefit brought about by the suspension culture was not consistently observed, leading the author to wonder whether adaptation to the suspension culture is strain-dependent [235].

Despite these initial doubts, subsequent studies have largely confirmed that suspension cultures yield not only a higher growth rate but also a lower number of multiply infected erythrocytes (i.e., the presence of two or more asexual parasites in the same erythrocyte) [236,237]. Allen et al. showed that a continuous suspension culture of synchronized parasites (reference clone 3D7) also resulted in longer maintenance of synchrony for three cycles compared to the static culture [237]. Furthermore, the suspension culture to initiate the cultivation of cryopreserved *P. falciparum* isolates was reported to result in higher multiplication rates than the static culture in 10 isolates [213]. A similar result (a four-fold reduction in the number of multiply parasitized erythrocytes) was reported using the rotation method (i.e., cultures in 15 mL polypropylene tubes that were tightly recapped after gassing with 3% O_2_, 5% CO_2_, and 92% N_2_ mixtures and rotated at 14 rotations per minute (rpm) at 37 °C in a hybridization incubator or oven [238].

Because erythrocytes are not in a monolayer but stacked in multiple layers in static cultures, the exchange of dissolved gases, nutrients, micro- and macromolecules, and waste metabolites between the culture environment and the cells may also be somewhat suboptimal compared to the suspension cultures. In spite of these disadvantages of static cultures, supported by experimental observations, static cultures, rather than suspension cultures, have become a common practice in most malaria research laboratories for practical reasons. Suspension cultures require suitable culture vessels, such as culture flasks or tubes that can be tightly recapped. An appropriate gas mixture may need to be flushed into the vessels before tight closure. Petri dishes are not suitable or at least may be difficult to handle on a shaker. A horizontal orbital (flask) shaker placed in an incubator occupies space. An alternative method developed by Trager and Jensen, the continuous flow method, shares some features of both suspension (constant flow of culture medium) and stationary (settled erythrocyte layer) methods, although the erythrocytes remain stationary [27]. Flow vials are not commercially available; only a few specialized laboratories in the world still use the flow vial method or its variants. The growth rate of laboratory-adapted parasites in static cultures is generally satisfactory for routine cultivation. Based on these practical considerations, static cultures have been preferred over suspension cultures for routine purposes.

Investigators requiring a large number of parasites for their experiments (for example, protein purification) may resort to the agitation of culture flasks to increase parasite yield. With suspension culture, care must be taken to minimize the physical damage to erythrocytes due to constant agitation. An addition of methylcellulose (1 mg/mL) to the culture medium has been suggested to protect the erythrocytes from mechanical damage, reducing hemolysis in suspension cultures [51]. Moreover, the type of medium displacement used is important. Vibratory suspension involving the minimum medium displacement was suggested to be optimal in early studies [53]. Presumably, this prevents the washing away of the released merozoites from the areas of cell concentration.

Although one of the basic principles of in vitro cultivation of malaria parasites involves a thin layer of erythrocytes bathed in a shallow volume of medium in a stationary set-up [27], “deep cultures” have been shown to be an alternative procedure for maintaining and producing a large number of parasites. Moloney et al. successfully cultivated laboratory-adapted strains in 5–15 L glass bottles containing a 3–8 L blood–medium–human serum mixture [239]. These deep cultures were maintained in constant suspension by a magnetic stirrer. To optimize parasite yield, the stirring speed needs to be increased with increasing volume and depth. Other variants of “deep cultures” developed more recently are discussed in the following section.

## 10. Tissue Culture Ware and Devices

Since the pioneering works of Bass and Bass and Johns [17,18], a wide variety of culture vessels, including glass tubes, Erlenmeyer flasks, flow vials, and others, have been used for the in vitro cultivation of malaria parasites. Glassware had been commonly used in the past for cell culture in general. Nowadays, plasticware (usually polystyrene or polycarbonate) has largely replaced glass for culture. Culture-grade plastics are treated to produce a wettable, charged surface, a property that is especially important for adherent cells.

For malaria cultivation, any sterile, flat-bottom, tissue-grade plastic (usually polystyrene) may be used. An open vessel (microtiter plate, Petri dish, tissue culture flask) is designed to equilibrate bicarbonate in the medium with gaseous CO_2_ in the incubator. An airtight closed vessel is more appropriate if pre-mixed gas is flushed into the vessel and placed in an incubator without high CO_2_ content. It is important to fill an appropriate volume of blood-medium mixture in each well, Petri dish, or culture flask to avoid the formation of a meniscus, which would result in suboptimal parasite growth [148].

In the original method developed by Trager and Jensen, both continuous flow vessels and Petri dishes were shown to be suitable [27]. Several semi-automated or manually operated perfusion or continuous flow devices were described and developed to propagate malaria parasites in the past [16,32,240,241,242,243,244,245,246,247]. These special culture devices, which are not commercially available, are too sophisticated for routine use in most laboratories and are not necessary for most research works, with the possible exception of the production of infective gametocytes. Depending on the purpose, *P. falciparum* can be cultivated in 6-, 24-, 48-, or 96-well microtitre plates, Petri dishes of various diameters, and tissue culture flasks of various sizes.

Since the early days of malaria cultivation, investigators have been attempting to adapt the basic methods of “small-scale” cultivation to produce large quantities of parasites for various experimental purposes, in particular biochemical studies [53,242,245,247,248,249]. For large-scale preparation of parasite material, Trager advocated the use of the flow vessel [250,251]. Some modifications, such as additional glucose in culture medium, suspension culture, and increased frequency of medium change, resulted in higher parasitemia, but limited volume posed a major technical difficulty.

The multiplication of culture vessels, for example, cultivation in several large culture flasks, would be cumbersome to handle. In the protocol developed by Radfar et al. [122], however, the number of 150 cm^2^ culture flasks (with up to 140 mL of culture medium per flask; RPMI 1640, 25 mM HEPES, 1.77 mM NaHCO_3_, 0.5% Albumax, 100 µM hypoxanthine; incubation in 5% CO_2_ incubator) at any one time is limited to three. High parasitemias (about 50%; hematocrit 0.8–1.5%) of synchronous cultures of laboratory-adapted reference clones (3D7 and Dd2) can be attained in three 150 cm^2^ flasks every four days, two of which are harvested, and the content of the third flask is divided and subcultured in three new 150 cm^2^ flasks. The culture medium is replaced once a day, and fresh erythrocytes (stored <14 days) are used for subcultures. It was reported that 1.2 mg of parasite protein per mL of harvested culture can be obtained in a week using this protocol.

To overcome the limit imposed by a relatively small volume of culture medium in traditional culture vessels, a method of deeply stirred cultivation using a large vessel (5–15 L glass bottles with 3–8 L of culture medium and a system of semi-automatic medium replacement) was described and shown to be feasible with a laboratory-adapted *P. falciparum* strain [239].

Over the past decades, several alternative techniques have been developed to maximize the yield of cultures of various cells. One of these is the three-dimensional hollow-fiber capillary bioreactor [252,253], which is also called “artificial capillaries”, originally developed for hemodialysis. This device consists of a cartridge containing thousands of hollow, semipermeable, capillary-like plastic tubules or fibers. Cells are inoculated in the space between the fibers. The culture medium is perfused by a pump over the large surface area per unit volume to provide maximal nutrient and gas exchange. Pressure is maintained and controlled by a microprocessor to ensure a homogenous medium flow and distribution.

In the first study that assessed bioreactor for malaria cultivation, Li et al. reported that this device allowed *P. falciparum* cultures with glucose- and hypoxanthine-supplemented RPMI 1640–HEPES–NaHCO_3_ media at high hematocrit (50–100%) [254]. The increase in the yield of infected erythrocytes with the hollow-fiber capillary bioreactor can attain 81-fold over 4–5 days compared with flask cultures. Using this culture system, a laboratory-adapted strain was maintained at the hematocrit of 50% for 16 days, with subcultures every four days. The maximum parasite density that can be attained with this device seems to be limited to 12% (>20% with a Petri dish or continuous flow method, depending on the hematocrit), but with 50–100% hematocrit, this would be desirable. A British–Thai research group reported that when using a laboratory-adapted mefloquine-sensitive Thai strain TM036, the total parasite yield in the bioreactor was equivalent to 19 T25 culture flasks (with a surface area of 25 cm^2^) on day 9 and 152 T25 flasks on day 35 of continuous culture [255,256]. In their study, 250 mL/day of a culture medium (RPMI 1640 supplemented with 10% human serum or 0.5% Albumax^®^ II + hypoxanthine + d-glucose; 40% hematocrit; change in the culture medium, once daily) was required.

A somewhat less sophisticated, single-use plastic bioreactor bag has been widely used in pharmaceutical and biotechnological sectors over the last two-to-three decades. The plastic bag is typically made of a multilayered high-quality polymer film. The bioreactors are usually designed for a suspension or shaking culture, with either a stirrer integrated into the bag or a support that produces a rocking motion [257,258]. The bioreactor was adapted for the cultivation of laboratory-adapted *P. falciparum* strains [259]. The parasites (3D7 and Dd2 strains) were grown in a bioreactor containing 1 L of medium (RPMI 1640 supplemented with 0.5% Albumax^®^ and 1 mg/L hypoxanthine; 2–4% hematocrit; equivalent of 20 T150 culture flasks) over 2–3 intraerythrocytic cycles. The bioreactor was placed on a rocking temperature-controlled support and was directly supplied with a gas mixture of 3% O_2_, 5% CO_2_, and 92% N_2_. The authors reported that the multiplication rate of the parasites in the control static culture flask and wave bioreactor was similar (starting parasitemia, 0.36%; final parasitemia after 3–5 days, 4–5%) and, as expected, multiply infected erythrocytes were observed more frequently in the static cultures.

The availability of research-grade biological samples, such as asexual blood-stage malaria parasites, that are safe and suitable for administration to human volunteers can facilitate the implementation and evaluation of clinical trials on vaccine candidates and novel antimalarial drugs [260]. Such *P. falciparum* cell banks have been produced over 16 days of continuous culture using a wave bioreactor (a final culture volume of 2 L in a 10 L cellbag; 6.3% parasitemia, synchronized to obtain 96% of the parasites at ring stage) [261].

Unlike “home-made” culture vessels used by a number of investigators, bioreactors are commercially available. Cultures are performed in a sterile, closed system. The closed system offers many advantages over the traditional methods of static and suspension cultures in Petri dishes or culture flasks, including higher parasite yield and less labor-intensive manipulation, but it requires a relatively high initial investment to acquire the apparatus (at least for hollow-fiber capillary bioreactors). These devices may be of interest to investigators requiring a large quantity of parasite material. One of the disadvantages of the large-scale cultivation system using bioreactors is that since they were not specifically designed for malaria cultivation, removal and replenishment of the culture medium and uninfected erythrocytes may have to be performed in a laminar flow hood. Some models may also require a setup inside an incubator with an appropriate gas mixture. Moreover, long-term cultivation of *P. falciparum* strains beyond 16 days in a bioreactor does not seem to have been performed, reported, or published.

## 11. Atmosphere

In the human host, O_2_ concentrations under normal physiological conditions vary widely in different parts of the body: 13.2–13.7% (partial pressure, 100–104 mm Hg) in the alveolar air and pulmonary capillaries, 12.5% (95 mm Hg) in the arterial blood, 5.3% (40 mm Hg) in the venous blood and interstitial fluid, and 0.6–5.3% (5–40 mm Hg) within the cells and tissues (for comparison, 20.95% O_2_ (159 mm Hg) in the atmospheric dry air). The diffusion of oxygen occurs from the alveolus (oxygen partial pressure, 100–104 mm Hg) into the erythrocytes passing through afferent pulmonary capillaries (partial pressure, 40 mm Hg) due to the difference in oxygen percentage composition. Most oxygen molecules in circulating blood (97%) are reversibly bound to the heme iron of the hemoglobin (up to four heme binding sites per hemoglobin molecule) and transported to the cells. A small proportion of oxygen (3%) is dissolved in the plasma. Oxygen is removed as blood passes through the tissues outside the lung, resulting in a low partial pressure of oxygen in the afferent pulmonary capillaries of 40 mm Hg in the venous end of the capillaries. Oxygen is used as the terminal electron acceptor of aerobic respiration in mitochondria in human tissues and passes back into the blood as CO_2_ and H_2_O.

The diffusion of carbon dioxide occurs in a direction opposite to oxygen. Carbon dioxide is produced by cells, is diffused into the local capillaries, and is transported by the blood to the lungs in three different forms: (i) bicarbonate ions (70%), (ii) dissolved CO_2_ in plasma (7%), and (iii) carbamino compounds, i.e., reversible binding of CO_2_ with hemoglobin and plasma proteins (23%). Carbon dioxide enters into the erythrocytes, and bicarbonate ions (HCO_3_^−^) are formed by the hydroxylation of CO_2_ catalyzed by carbonic anhydrase that is present in the erythrocytes but not plasma. HCO_3_^−^ diffuses out of the erythrocytes, and chloride ions enter the erythrocytes to maintain the ionic balance (a phenomenon called the chloride shift). H^+^ ions are buffered by hemoglobin. Unlike O_2_, CO_2_ concentrations vary only slightly (range, 5.3–6.1% (40–46 mm Hg); for comparison, 0.03–0.04% (0.2–0.3 mm Hg) in the atmospheric dry air) in different organs and tissues of the human body under normal physiological conditions, but the slight difference is enough to ensure CO_2_ diffusion and transport from the peripheral tissues to the lungs. The remaining gases in the human body are mostly composed of N_2_ (74.9–75.4% (569–573 mm Hg); for comparison, 78–79% (594–601 mm Hg) in atmospheric dry air) and water vapor (6.2% (47 mm Hg)) at sea level at 37 °C.

Malaria parasites are obligate microaerophilic organisms, i.e., the parasites require O_2_, but at low concentrations. They do not grow under strictly anaerobic conditions. At the other extreme, the normal O_2_ content (20.95%; partial pressure, 159 mm Hg) in the atmosphere has been suggested to be too high for optimal parasite growth. In the human host, erythrocytes infected with ring-stage *P. falciparum* parasites passing through the lung capillaries may be exposed to 13–14% O_2_ (partial pressure, approximately 100–104 mm Hg). However, in the venous end of the capillaries of deep organs where erythrocytes harboring *P. falciparum* trophozoites and schizonts are sequestered, O_2_ concentration falls to approximately 5% (partial pressure, 38 mm Hg). A low O_2_ tension in an incubator mimics the in vivo conditions to which human malaria parasites are naturally subjected. Therefore, it is not surprising that a microaerophilic environment favors parasite growth.

The understanding of what is now basic fact was one of the key experimental factors that eventually led to the development of continuous culture. Trager and Jensen developed the flow vial method in an atmosphere of 7% CO_2_, 5% O_2_, and 88% N_2_ [27]. Haynes et al. developed their culture system in an atmosphere of 3% CO_2_–6.6% O_2_ [26]. An alternative method developed by Trager and Jensen, called “the candle jar method”, requires a simple, inexpensive air-tight device in which cultures in Petri dishes or culture plates are placed, and a candle is lit to consume O_2_ within the device [27]. When the candle goes out, an atmosphere of approximately 3% CO_2_, 17–18% O_2_, and 79–80% N_2_ is created within the candle jar [27,81,107,115,262] (Table 4).

The first study on the various combinations of O_2_–CO_2_ gas mixtures showed that the optimal growth of a *P. falciparum* strain occurs in a mixture of 2–3% O_2_, 1–2% CO_2_, and 95% N_2_ over a 96–144 h incubation period [107]. The parasite strain (FCR-3/The Gambia) also grew in a 5% CO_2_ incubator (about 19–20% O_2_) at a similar multiplication rate as the 3 or 5% O_2_–5% CO_2_ mixture or in a candle jar (16.1–17.8% O_2_ and 1.1–2.1% CO_2_) for up to 72 h. The multiplication rate in 5% CO_2_ in the air decreased to about 50% of that attained in the 3 or 5% O_2_–5% CO_2_ mixture or in a candle jar after 96–120 h of incubation. The growth rates at the microaerophilic level of 0.5% O_2_ were generally similar to, or slightly less than, those with 1–5% O_2_, depending on the CO_2_ level and the duration of incubation. The gas mixture of 3% CO_2_ in the air and the total anaerobic atmosphere did not support parasite growth. In another study, parasite growth assessed for three days was shown to be similar with two different gas mixtures (5% O_2_–5% CO_2_ versus 10% O_2_–5% CO_2_) and in a candle jar (about 17–18% O_2_–3% CO_2_), but O_2_ content <5% was reported to be inhibitory [29].

As reported by Scheibel et al. [107], other authors have also suggested that 5% CO_2_ in the air (i.e., approximately 19–20% O_2_–5% CO_2_) may not support the growth of malaria parasites in vitro. Butcher reported that a laboratory-adapted strain (HG13/The Gambia) grew for one or two intraerythrocytic cycles in 5% CO_2_ in the air, but the normal atmospheric content of O_2_ inhibited the growth of the parasites beyond two intraerythrocytic cycles [29]. Similar findings were reported by Binh et al. [167] in a single laboratory-adapted isolate (BINH/Kenya) maintained in a serum-free medium and cultivated in 5% CO_2_ in the air. On the contrary, other investigators have found that the atmosphere of 5% CO_2_ in the air (in the CO_2_ incubator) is suitable (but not always optimal compared to cultures in a gas mixture of 5% O_2_, 5% CO_2_, and 90% N_2_), for both the short-term and long-term cultivation of freshly isolated parasites and laboratory-adapted *P. falciparum* strains [103,106,265].

The incubation of laboratory-adapted strains (3D7 and W2) in the 21% O_2_, 5% CO_2_, and 74% N_2_ gas mixtures may have an adverse effect on parasite growth [266,267]. Morphological alteration was not observed under an optical microscope compared with the controls (5% O_2_, 5% CO_2_, and 90% N_2_; 10% O_2_, 5% CO_2_, and 85% N_2_ gas mixtures), but parasitemia decreased in 3D7 during the second intraerythrocytic cycle when exposed to 21% O_2_. The authors also noted that the duration of schizogony was prolonged in parasites (mature trophozoites and schizonts) exposed to 21% O_2_, leading to a 4 h delay in the schizogonic phrase of 3D7 (45 h cycle in 5% O_2_ versus 49 h cycle in 21% O_2_). This modification was due to the later initiation of nucleic division, as evidenced by the delayed appearance of schizonts. Short-term cultivation (two intraerythrocytic cycles) of 3D7 in 10% O_2_ had no significant effect on the duration of the intraerythrocytic cycle, parasitemia, or parasite stages compared to the control culture in 5% O_2_.

In another study, the growth of parasites (3D7 clone) cultivated in 20% O_2_, 5% CO_2_, and 75% N_2_ for 48 h was reported to be similar to the control parasites cultivated in 5% O_2_, 5% CO_2_, and 90% N_2_, but in the former condition (20% O_2_), vitamin E biosynthesis of the parasites increased significantly and the level of lipid peroxidation decreased, protecting plasma membranes from damage due to reactive oxygen species (ROS) (α-tocopherol reduces the amount of ROS produced during oxidative stress) [268]. The study suggested that a relatively high O_2_ content may not necessarily lead to deleterious biochemical effects, but the effects of 20% O_2_ were not assessed beyond 48 h.

In the experiments conducted by another research group, both the oxygen concentration (5% O_2_, 5% CO_2_, and 90% N_2_ versus 21% O_2_, 5% CO_2_, and 74% N_2_) and the type of serum supplementation (pooled human serum (5% *v*/*v*) with Albumax^®^ II (2.5 mg/mL) versus Albumax^®^ II alone (5 mg/mL)) used to cultivate 3D7 clones for several months influenced the duration of intraerythrocytic cycle in vitro. The duration ranged from a 40 h cycle for 3D7 cultivated with the serum Albumax^®^ II mixture supplemented to the standard RPMI 1640 medium in 5% O_2_ to a 44 h (3D7 cultivated with Albumax^®^ II, without human serum in 21% O_2_) or a 45 h cycle (3D7 cultivated with Albumax^®^ II, without human serum, in 5% O_2_) [215].

Contrary to the general consensus among malaria researchers that an optimal long-term in vitro cultivation of *P. falciparum* requires low O_2_ concentration (i.e., any value slightly above anaerobic condition and that obtained in a candle jar or 5% CO_2_ incubator), a research group reported that several *P. falciparum* reference strains have been maintained in a normal atmospheric gas mixture (20.95% O_2_, 0.04% CO_2_, 78–79% N_2_) in their laboratory for several months to one year in a standard RPMI 1640 medium with 0.5% Albumax^®^ supplemented with hypoxanthine and 2 g/L glucose [269]. The suspension cultures (starting parasitemia > 0.5%) were maintained in culture flasks (with the cap screwed tightly) or hermetic glass bottles (0.1–2 L bottles; 20% of the volume was filled with culture medium and 80% of the volume was occupied by atmospheric gas or the standard gas mixtures of 5% O_2_, 5% CO_2_, and 90% N_2_; the latter was flushed into the culture vessel before screwing the cap) placed on an orbital shaker. When suspension cultures in atmospheric gas and the standard gas mixture were compared, parasite growth during six days was similar. The parasites in static cultures in glass bottles did not grow. The free oxygen contents in uninfected erythrocytes measured after 24 h in the flasks with atmospheric air or the standard gas mixture were 180 µM and 147 µM, respectively (arterial oxygen concentration, 130 µM).

In an earlier experiment, the same research group reported that a reference clone (3D7) was cultivated in their laboratory in an anaerobic condition (i.e., 5% CO_2_ and 95% N_2_) for 10 days, and the multiplication rate remained similar to the control parasites cultivated in the standard gas mixture (5% O_2_, 5% CO_2_, and 90% N_2_), leading the authors to conclude that the parasite adapted to anaerobic conditions [270]. Details of parasite growth were not described in that study.

Another effect that O_2_ (or CO_2_) concentration may have in short-term cultivation of malaria parasites is possible changes in the level of susceptibility in vitro to some antimalarial drugs and antibiotics [215,262,266,269,271,272]. Transcriptomic data suggest various metabolic changes in response to hyperoxia [267], but the precise underlying reason for the influence of O_2_ concentration on the level of in vitro drug susceptibility is not yet well-known. In vitro response to chloroquine is also influenced by CO_2_ concentration; chloroquine IC_50_ decreases significantly in a chloroquine-resistant laboratory-adapted strain (K1/Thailand) when CO_2_ concentration varied from 7% to 5% and 2.7% with a constant O_2_ concentration set at 5% [262]. Growth rates in the drug-free control wells were not affected by CO_2_ concentration during the 42 h incubation period. The underlying cause of altered IC_50_ values is due to pH modification (a higher CO_2_ concentration leads to lower pH of culture medium) resulting from an imbalanced CO_2_-NaHCO_3_ buffer system. These observations have important implications for data interpretation of in vitro drug response.

The candle jar method is practical and affordable in most laboratories, including those in the field. The candle jar still needs to be placed in a simple incubator to maintain it at 37 °C. The possibility of using an ordinary CO_2_ incubator, rather than using the more expensive, sophisticated incubator with O_2_ regulation, for malaria cultures, including short-term cultures (e.g., for in vitro drug susceptibility assays requiring 24–72 h of incubation), is an important option for moderately equipped field laboratories. Normal air, however, is generally not suitable for malaria cultivation, even for laboratory-adapted *P. falciparum* strains [273]. For fresh clinical isolates, some investigators noted that the use of a commercial gas mixture of 5% CO_2_–5% O_2_–90% N_2_, instead of a candle jar, allowed a higher proportion of isolates to be adapted to long-term in vitro culture conditions [274].

In addition to the use of a candle jar, the normal gas composition in the atmosphere can be modified by (i) using an incubator that can be programmed to regulate the CO_2_ and/or O_2_ contents and placing Petri dishes or tissue culture flasks with vented caps (or, if they do not have vented caps, place the cap loosely on the flasks) or (ii) flushing pre-mixed gas into culture flasks before replacing the cap tightly. With both of these methods, cylinders containing pure CO_2_, pure N_2_ (to lower O_2_ content), or various mixtures of CO_2_, O_2_, and N_2_ (pre-mixed gas) are required. For maintenance, a 5% CO_2_ incubator requires a single source of gas (CO_2_), and it is less expensive than the more sophisticated incubator with adjustable levels of O_2_ and CO_2_, for which two sources of gas, CO_2_ and N_2_, are required. If a CO_2_ incubator, with or without variable O_2_ control, is not available, pre-mixed gas can be used, but it is often unavailable in many malaria-endemic countries. Ordering pre-mixed gas from an industrialized country would be costly and would take time. One possible solution to this problem would be to use an adjustable gas mixing device [275]. Such a device still requires a relatively high initial investment to acquire and install the gas supply system. Gas cylinders with pure CO_2_, pure N_2_, and pure O_2_, which may be available locally, are also necessary to operate the system.

Several less sophisticated methods to modify the gas contents for malaria cultivation have been developed. One simple method that does not require any additional equipment is to blow end-tidal-exhaled air into the culture flask [276]. Tidal volume refers to the amount of air inspired and expired with each breath during quiet respirations, i.e., without any added effort during inspiration or expiration. At the end of expiration, the alveolar partial pressure of O_2_ increases and lowers the partial pressure of CO_2_ to a minimal level. To obtain a suitable gas composition in exhaled air, a person inhales air normally without effort, holds the breath for 15 s, and exhales into the culture flask through a sterile cotton-plugged Pasteur pipette. Immediately after expiration, the flask is tightly sealed. The gas composition of end-tidal-exhaled air was found to contain 5.1% CO_2_, 15.5% O_2_, and 78% N_2_, which are close to the gas mixture in a candle jar. Using end-tidal-exhaled air as the source of gas, a laboratory-adapted *P. falciparum* strain was successfully maintained for 29 days. The growth rate was similar or slightly lower with end-tidal-exhaled air than pre-mixed gas (5% CO_2_–5% O_2_–90% N_2_) for the first 14 days and tended to be about three-to-four-fold lower than pre-mixed gas between day 14 and day 29 of cultures.

Another method involves the introduction of a single-use chemical pack containing ascorbic acid within an airtight culture vessel. Once opened, these packs absorb O_2_ from the enclosed air and release CO_2_ in a chemical reaction and modify the atmosphere to one of the following pre-determined gas composition, depending on the type of gas sachets (Sugiyama-gen, Tokyo, Japan): (i) 5% CO_2_ and 15% O_2_, (more adapted for bacterial culture; similar to a candle jar), (ii) 10% CO_2_ and 10% O_2_, or (iii) 5% CO_2_ and 5% O_2_ (specifically manufactured for malaria culture). The specified atmosphere is reported to be attained within 2 h in a hermetically sealed container and can be maintained for over 24 h.

Preliminary studies have demonstrated that both fresh clinical isolates and laboratory-adapted *P. falciparum* strains can be cultivated with this novel system [277,278,279,280]. The growth rate of laboratory-adapted parasites was similar in three different gas mixtures produced by the sachets, i.e., the 1–3% O_2_/3–4% CO_2_/93–96% N_2_ gas mixture, and a candle jar over a 48 h period. Moreover, the long-term cultivation of laboratory strains was successful for 26 days at similar growth rates in different gas mixtures. In vitro drug susceptibility assays can also be performed in the field with these gas-generating sachets [281,282,283,284,285].

The general consensus among malaria researchers is that, for the long-term growth of parasites under optimal conditions, O_2_ level should be lower than 21% found in the air. Until today, however, there is no consensus as to what should be the actual O_2_ concentration for malaria cultivation [286,287]. According to Jensen and Trager, the actual O_2_ level is not critical for malaria cultivation, and it may range from 5 to 17%, with 10–15% giving the best results [81]. More recently, other authors have argued that O_2_ levels between 1 and 5% should be considered as the physiological normoxic O_2_ range for asexual stages of malaria parasites in the human host [287]. During the ring stage in vivo (for about 24 h), *P. falciparum* parasites come in contact with relatively high O_2_ (>13%) levels for a brief moment while in transit in the pulmonary circulation and circulate during the rest of the time in arterial (12.5% O_2_), venous (5.3% O_2_), and capillary blood (see Table 4). The mature *P. falciparum* trophozoites and schizonts spend the next 24 h sequestered in deep organs, where oxygen concentration falls to 0.6–5.3%. Based on human physiology and the fact that *P. falciparum* has adapted to fluctuating O_2_ levels in the host, mostly to oxygen tension below that found in pulmonary capillaries, it can reasonably be argued that the levels of O_2_ ranging from 0.6% to 13% in in vitro cultivation probably best mimic the in vivo condition.

For CO_2_, even though its concentration can be as high as 5.9–6.1% in the deep venous circulation and at the tissue level (see Table 4), levels >5% CO_2_ are too high for the CO_2_ bicarbonate buffer system to maintain optimal pH. In vivo, the range of CO_2_ concentration is narrow. The standard medium containing RPMI 1640 and the double buffer system, 25 mM HEPES and 25 mM NaHCO_3_, was conceived to maintain the pH within the physiological range in an atmosphere with 5% CO_2_. The percentage of CO_2_ in the atmosphere influences the pH of the culture medium depending on the buffer system. A relatively high (5%) CO_2_ is required to maintain the pH of the RPMI 1640–HEPES–NaHCO_3_ medium to approximately 7.2. At lower (1–2%) CO_2_, the pH of the medium rises to 7.5–7.7. For a given CO_2_ concentration, a wide range of O_2_ content between 0.5% and 21% does not modify the pH. The optimal CO_2_ level for in vitro cultivation of *P. falciparum* is probably 3–5%.

Most strains of *P. falciparum* grow within a wide range of O_2_ levels, but at higher levels (17–21%), the growth rate tends to decrease unless the CO_2_ level is raised to 5%. The requirement for low O_2_ may be strain-dependent. Some strains, in particular chloroquine-susceptible parasites, may require a lower O_2_ level (3–5%) and/or human serum (instead of serum-free substitutes, such as Albumax^®^) for optimal long-term cultivation. Researchers may have to check if *P. falciparum* strains in their laboratory are well-adapted to certain O_2_ and CO_2_ concentrations.

In a recent experimental work conducted by Géry et al. [288], the authors reported successful long-term cultivation of a laboratory-adapted *P. falciparum* clone (3D7) using a Petaka G4™ device (Celartia, Columbus, OH). This relatively simple, compact, and light cell culture vessel with an auto-controlled gas exchange system was designed to cultivate various cells without any internal air space. The vessel is filled with complete culture media and parasite-infected erythrocytes, leaving no air space within the vessel. Minimal gas exchange with external air occurs through its 0.2 µm filter. The removal of the spent medium and the introduction of fresh media and uninfected erythrocytes are performed through its injection port. Due to its completely closed system, many of the routine steps involved in malaria cultivation can be performed outside a laminar flow hood. It could be hypothesized that a small proportion of O_2_ that is dissolved in the plasma and available in hemoglobin molecules may be sufficient for *P. falciparum* parasites to propagate in this closed system. Although further studies are necessary, this novel system to cultivate malaria parasites provides an avenue worth exploring to understand the O_2_ and CO_2_ requirements of malaria parasites.

## 12. Incubator

The requirement for an incubator or another piece of equipment, such as a water bath or temperature-controlled orbital shaker, that maintains a constant temperature of 37 °C for at least 24–72 h (to perform in vitro drug susceptibility assays or initiate in vitro cultivation) is one of the limiting factors for a wide application of malaria cultures in the field. In developed countries, almost every microbiology laboratory possesses an incubator. It may be a simple incubator with normal atmospheric air or a more sophisticated incubator with 5% CO_2_ in the air, or it may have options to adjust both CO_2_ and O_2_ concentrations. In developing countries, even if an incubator were available, the power supply may not be reliable in some endemic areas due to frequent power cuts and voltage fluctuations.

Few attempts have been made to develop portable incubators designed for field use. A “home-made” portable incubator heated by a car taillight bulb and powered by a car battery was designed and tested by Eastham and Rieckmann [289]. An oven thermostat is connected to the circuit to switch the electric bulb on and off to maintain the temperature at 37 °C. A candle jar desiccator made from large glass jars that can accommodate microtiter plates is placed in the incubator. Such a device may be useful in a remote field site where there is no source of electricity.

Another option is offered by a Japanese manufacturer that has developed a portable incubator in which culture plates or Petri dishes and gas-generating ascorbic acid sachets (5% CO_2_–5% O_2_ or 5% CO_2_ in air) are placed within an airtight box (AnaeroPack^®^ malaria culture system; Sugiyama-gen Co., Ltd., Tokyo, Japan) [279,283,284]. The incubator requires a constant electrical supply source, which may be from a car battery. The available model functions on 110 V (no built-in transformer for countries with 220 V) and is relatively expensive.

Where there is at least a power line, other devices may be used. Water baths, which can be found in most laboratories, may serve as an incubator [290]. If a field-compatible incubator, water bath, or temperature-controlled orbital shaker is not available for on-site in vitro assays, blood samples would have to be transported to the nearest research laboratory.

## 13. Temperature

Despite the fact that parasites may be subjected to a relatively high temperature (>40 °C) in the human host, at least for a few hours during febrile episodes, in vitro experiments have suggested that prolonged hyperthermia (i.e., continuous exposure to ≥40 °C for 24–72 h) may actually be deleterious for parasite growth [291,292,293,294]. In the experiments conducted by Kwiatkowski [291], it was observed that laboratory-adapted parasite strains (ring stage) exposed to 40 °C during the first half of the intraerythrocytic cycle (i.e., 0–24 h), followed by cultivation at 37 °C for the remaining half of the intraerythrocytic cycle (i.e., 24–48 h), grew almost as well as the control parasites cultivated at 37 °C for 48 h. By contrast, when the parasites were cultivated first at 37 °C for 24 h, followed by incubation at 40 °C for the next 24 h, the 48 h parasitemia decreased considerably compared to the control parasites cultivated at 37 °C for 48 h, and pyknotic schizonts were observed. This earlier finding was not confirmed in a more recent study. In the experiment conducted by Singhaboot et al. [294], even a short 2 h exposure of a laboratory-adapted strain in the ring stage to 40 °C, followed by culture at 37 °C for the next 46 h, led to not only a lower multiplication rate but also morphological alteration, suggesting parasite death. Those authors have reported that cultivation at 38 °C and 39 °C continuously for 48 h also decreases the multiplication rate (roughly 50% of the multiplication rate of the control parasites incubated at 37 °C), but there was no significant difference in the multiplication rates between 38 °C and 39 °C [294]. Exposing the parasites to 38 °C or 39 °C during the first 2 h of the intraerythrocytic cycle followed by incubation at 37 °C for the rest of the first intraerythrocytic cycle did not affect the multiplication rate measured at the end of the first intraerythrocytic cycle. In the experiments conducted by Long et al., it was shown that if the parasites are incubated continuously at a high temperature (38–39 °C), parasite growth decreases significantly at 38.5 °C and 39 °C but is not affected to a significant extent at 38.0 °C [292]. Some of these experimental studies have shown that ring-stage parasites are relatively more thermoresistant than mature trophozoites and schizonts [291,292].

A more elaborate experimental design involving the exposure of *P. falciparum* ring-stage cultures to 40 °C for 2 h (first heat shock) and the recovery phase at 37 °C for 10 h followed by a second exposure to 40 °C for 24 h (second heat shock; then incubation at 37 °C during the second intraerythrocytic cycle) showed that the growth of the parasites exposed to these two phases of heat shock was not affected during the first intraerythrocytic cycle and increased significantly during the second intraerythrocytic cycle [295]. The control cultures maintained at 37 °C and exposed to the second heat shock, without being exposed to the first heat shock, resulted in a marked diminution in parasite growth during the first intraerythrocytic cycle. The authors concluded that exposure to a short period of high temperature protects the parasites from subsequent heat shock and results in a higher multiplication rate. The clinical implications of the in vitro experiments described above are not clear.

When laboratory-adapted *P. falciparum* parasites are cultivated continuously at low temperatures (28–34 °C) for 48–72 h, maturation is considerably delayed, and/or the multiplication rate is considerably reduced [293,294,296]. A short 2 h exposure of ring-stage parasites to 32–35 °C followed by incubation at 37 °C for 48 h does not significantly affect the parasitemia level or morphology of the parasites [294]. At a much lower temperature (0–4 °C), the growth of laboratory-adapted *P. falciparum* strains is completely arrested. Mature developmental stages (i.e., mature trophozoites and schizonts) are killed when kept at 0–4 °C for 24–48 h [297,298]. Ring-stage parasites may remain viable for up to 14 days at 4 °C; when replaced in suitable culture conditions at 37 °C, in vitro cultivation can be pursued [299]. For fresh blood samples, many field investigators have been transporting samples obtained from malaria-infected patients in remote areas to research laboratories in a cooler or insulated box with cold packs (gel packs or ice packs) or wet ice, which usually maintains the inside temperature within the range of 2–8 °C, depending on the ambient temperature, container, and the number of cold packs or the amount of wet ice in the box. Many, but not all, fresh clinical isolates of *P. falciparum* can be preserved in wet ice for up to 10 days before initiating in vitro cultivation or ex vivo drug susceptibility assays [300].

The first successful continuous cultivation of asexual erythrocytic stages of *P. falciparum* was attained at an incubation temperature of 37–38 °C [26,27]. It has been, and still is, recommended to maintain in vitro cultures at 37 °C for routine maintenance of asexual *P. falciparum* parasites.

## 14. Axenic Culture

Axenic culture is a pure culture without any “contaminating” cells. It may be considered as an ultimate goal to be attained in most, if not all, cultivation procedures. In the case of asexual intraerythrocytic stages of malaria parasites, axenic culture means the propagation of the parasites extracellularly, without the presence of intact erythrocytes. In some early works on non-human malaria parasites, investigators have attempted to remove the asexual parasites from their host erythrocytes for cellular and biochemical studies [16,301,302,303]. The parasites survived and developed for up to four days, but there was no propagation.

Axenic culture of the erythrocytic stages of *P. falciparum* has been attempted by William Trager with limited success since the 1970s [16]. In the initial studies, schizonts incubated for 20–24 h in a modified, high-potassium RPMI 1640 medium supplemented with human red cell extract, human serum, ATP dipotassium salt, and sodium pyruvate (without intact erythrocytes) developed into extracellular young trophozoites [304]. In later studies, axenic culture initiated from both purified merozoites and young rings freed by lysis from their host erythrocytes resulted in developing extracellular late rings and trophozoites after 14–18 h using a similar culture medium to their earlier studies [305,306]. These extracellular trophozoites had essentially the same ultrastructure as the intracellular trophozoites.

Further improvement in the axenic development of one complete asexual erythrocytic cycle (and the early development of extracellular gametocytes) was attained by embedding purified merozoites in a gelatinous matrix overlaid with high-potassium RPMI 1640 supplemented with human red cell extract, serum, ATP, and sodium pyruvate [307,308,309]. As many as 30% of embedded merozoites differentiated into extracellular trophozoites, but only 1% reached the schizont stage after 36 h. The addition of human erythrocyte membranes (i.e., erythrocyte ghosts prepared by hypotonic lysis) increased the yield of viable extracellular trophozoites and early schizonts at 36 h. Early stages of gametocytes (stages II and III) were also observed. Merozoites produced by the extracellular schizonts during the first intraerythrocytic cycle of axenic culture were viable.

In the final study on axenic culture conducted in William Trager’s laboratory before its closure in 1998, further improvement was obtained with the addition of carnitine (100–200 µM), magnesium sulfate (10 mM), calcium nitrate (0.4 mM), GF21 serum substitute (10–20%), and higher erythrocyte membrane content (in addition to supplements such as 2.1 mM ATP, 5.4 mM sodium pyruvate, and 100 µM coenzyme A sodium salt used in earlier experiments [307,308,309]) and incubation in 0.5% O_2_, 3% CO_2_, and 96.5% N_2_. These modifications allowed three cycles of schizogony over a six-day period, with a five-fold increase in the number of parasites after each complete cycle [310]. When the extracellular schizonts obtained at the end of the experiment on day 6 were cultivated with intact uninfected erythrocytes, intracellular rings developed on day 7, demonstrating the viability and infectivity of parasites that developed extracellularly for three erythrocytic cycles. The experimental conditions need further improvements to ensure a consistently high yield of viable, extracellular parasites after each successive erythrocytic cycle in the continuous culture. Some clues for such improvements have been suggested by William Trager for future researchers [311]. At present, the continuous axenic culture of the erythrocytic stages of malaria parasites is still in its infancy.

## 15. Adaptation, Selection, and Phenotypic/Genotypic Modifications during Cultivation

The in vitro cultivation of *P. falciparum* has become a major tool for malaria research. Many concrete examples can be given to highlight the important roles that in vitro cultivation has played since 1976, including drug discovery, validation of candidate molecular markers of drug resistance, a better understanding of malaria biology, and vaccine development, to mention a few [13]. Yet, in our present state of knowledge, many unanswered questions about its relevance persist. Several “epistemological” questions (i.e., what and how can we know using *P. falciparum* cultivated in vitro?) have been raised by some authors, the writings (reviews and opinion papers) of whom readers are encouraged to peruse for reflection and debate [57,287,312,313,314]. Some of these questions are discussed below.

First and foremost, it should be borne in mind that *P. falciparum* parasites maintained in continuous in vitro cultivation do not faithfully represent naturally occurring parasites in the human host. The latter, referred to as an “isolate” when it is collected, is defined as “a sample of parasites, not necessarily genetically homogeneous, collected on a single occasion from a wild host and preserved in the laboratory either by passaging or by deep freezing” [315]. Early studies have shown that *P. falciparum* isolates or uncloned laboratory-adapted strains are often composed of several distinct parasite populations, each of which is characterized by an enzyme variant (or electrophoretic form) or genetic profile [316,317,318,319,320,321,322,323,324]. Molecular studies conducted during the 1990s on field isolates fully support these findings and have quantified the number of distinct parasite populations in an isolate obtained from individual human hosts (asymptomatic carriers or symptomatic patients), referred to as the “multiplicity of infection” (MOI) or the complexity of infection (COI) [325,326,327,328,329,330]. The average MOI in a patient population or asymptomatic carriers usually reflects the level of intensity of malaria transmission; it is high in areas characterized by perennial and intense transmission, as in many sub-Saharan African countries [331,332,333,334].

The first “barrier” to in vitro cultivation encountered by the parasite populations constituting an isolate is adaptation. Many, but not all, clinical isolates collected from different parts of the world can be adapted to short- and/or long-term cultivation using the standard methods described by Trager and Jensen [109,114,198,199,335,336,337,338,339]. Some of the parasite populations grow readily and adapt with ease to the new environmental and nutritional conditions, while others tend to become minority populations or even disappear (do they die out because the predominant populations use up the available resources or because they are inherently incapable of adapting to in vitro conditions, or do they persist at an undetectable level?). A recent study provides further evidence that, after adaptation to in vitro cultivation, one of the parasite populations in an isolate may become predominant over others and “impose” its phenotype, such as the multiplication rate, on others, masking the phenotypes of the minority parasite populations [340]. While this is an expected result, the opposite effect may be produced in some cases. The same authors also observed that clones derived from the original isolate displayed higher multiplication rates than the original uncloned isolate, possibly due to competitive suppression within the uncloned isolate, the presence of an undetected slow-growing parasite population in the uncloned isolate, or the selection of slow-growing populations with better fitness in the uncloned isolate.

In the literature, the term “culture-adapted isolate” or “culture-adapted strain” has been used without a clear definition. An isolate that has adapted to continuous in vitro cultivation may be loosely referred to as a new “strain”. However, the definition of a “strain” has not been established in a more stringent manner [341]. Jensen and Trager have observed that isolates generally take four to six weeks of continuous cultivation to become adapted and “stabilized” and, based on a small number of isolates in their experiments, isolates that are cryopreserved during continuous cultivation can be readily recovered beyond five or six weeks [335]. Some authors have proposed that a parasite is considered to be adapted to culture conditions when a multiplication rate of >10 every 96 h is attained [114]. Others have recommended that culture-adapted malaria parasites should refer “specifically to isolates of the parasite that have been grown continuously in vitro for at least 2 months and have been frozen, thawed, and successfully recultured at least once” [342]. Based on these three criteria (i.e., duration (at least four weeks), multiplication rate, and recovery after cryopreservation), Jensen and Trager have argued that the cultivation method developed by Haynes et al. [26], terminated after 22 days, was “abandoned too soon to permit any conclusion as to whether their conditions would in fact support continuous culture” [335]. From the pragmatic viewpoint, *P. falciparum* parasites cultivated in vitro (which no longer undergo sexual reproduction) that meet the aforementioned criteria may be said to constitute a strain, defined as “a homogeneous population possessing a set of defined characters” [341]. When these defined characters are based on genetic uniformity, which is attained in the laboratory by separating a single parasite-infected erythrocyte by dilution/microscopic selection [319], limiting dilution [158,343,344,345,346,347,348], micromanipulation [349,350], or single-cell sorting [351,352] and propagating it in vitro, the strain becomes a clone. Clonality is usually evidenced by the absence of polymorphisms in well-studied antigens, such as merozoite surface antigen-1 (MSA-1) and merozoite surface antigen-2 (MSA-2).

The capacity of parasite populations to adapt to in vitro conditions may have a genetic basis. A recent study has suggested that mutations in *P. falciparum* regulator genes (*ApiAP2* transcription factor genes, *Epac* gene encoding a guanine nucleotide exchange factor, *SRPK1* (serine/threonine protein kinase) genes, and *DOC2* (double C2-like domain-containing protein) genes) may be associated with culture adaptation [353]. In another series of experiments involving bulk segregant analysis, erythrocyte-binding antigen-140 (*eba-140* (note: it is one of the merozoite ligands that plays a key role in the entry of merozoite into erythrocyte by binding to the erythrocyte receptor, glycophorin) [354]), and, to a lesser extent, aspartate transaminase (*ast*) and cysteine protease autophagy-related 4 (*atg4*) genes, were identified as the key loci that determine fitness (i.e., parasite growth rate) in parasites grown in RPMI 1640 supplemented with either human serum or Albumax^®^ [209]. Other investigators have studied the expression patterns of clonal multigene families (or clonally variant gene families), such as *eba-140*, cytoadherence-linked asexual genes (*clag*), variant genes (*var*; encoding *P. falciparum* erythrocyte membrane protein 1, PfEMP-1), repetitive interspersed families (*rif*), and subtelomeric variable open reading frame (*stevor*) genes, which confer phenotypic diversity to genetically homogeneous parasite populations, which in turn may confer biological advantages, especially fitness, to the parasites to allow adaptation to different environments [355,356]. There is still limited experimental evidence that supports these recent findings. More studies are required to further understand whether there is a single set of genetic modifications associated with adaptation to in vitro conditions.

Continuous cultivation of mixed parasite populations usually, but not always, results in a diminution of the initial MOI and may lead to a selection of the dominant parasite population, possibly endowed with biological fitness [357,358]. For unknown reasons, several parasite populations initially present in the day 0 isolate may also continue to co-exist in vitro, and a “new parasite population” undetected by the polymerase chain reaction (PCR) in the initial isolate on day 0 may emerge within a short time period (i.e., within 9 days of continuous cultivation or four asexual intraerythrocytic cycles) [359]. In a study based on microsatellite genotyping, single nucleotide polymorphisms (SNPs) of drug resistance markers and phenotypic profiles (drug response), it was reported that three fresh Kenyan clinical isolates of *P. falciparum* exhibited various genotypic and phenotypic profiles, without any pattern or phenotype–genotype correlation, during the 2–3 month cultivation period [360]. In that study, the stabilization of the genotypic or phenotypic profile was not observed, and there was no apparent tendency toward the predominance of a distinct parasite population over the other parasite populations present in the isolate. These observations suggest that adaptation of *P. falciparum* parasites to in vitro conditions is a complex phenomenon resulting from (and/or leading to) biochemical and genetic modifications, which are in large part still unfathomed.

Once the first hurdle is cleared and the parasites become adapted to in vitro cultivation, or even during the adaptation period, the selection of parasite population(s) may or may not occur. As emphasized by Thompson and Lymbery [341], “a strain may include one or more populations”. The accumulated experimental evidence has demonstrated that, during the first few days or weeks of continuous in vitro cultivation, phenotype changes occur in many *P. falciparum* isolates, including drug response, cytoadherence, and multiplication rates [336,361,362,363].

After or during adaptation and selection processes, further phenotypic and/or genotypic modifications may also occur in laboratory-adapted *P. falciparum* strains or even clones. Phenotypic changes that can be induced by altering in vitro culture conditions were examined in detail in earlier sections. Briefly, such changes include multiplication and invasion rates, duration of the intraerythrocytic cycle, drug response, transcriptomic profile, the number and size of knobs, the number of variant surface antigens, and cytoadhesion to endothelial receptors. The major factors that have been shown to influence the phenotype include serum supplement (human serum versus Albumax^®^), O_2_ level, suspension culture, and hypoxanthine supplement. Some of these phenotypic changes may occur due to the cultivation conditions that the parasites encounter, i.e., they may be induced, knowingly or unknowingly, by investigators themselves. Genotypic changes likewise occur spontaneously during long-term cultivation in laboratory-adapted strains and clones, both at nucleotide and chromosome levels, which may or may not be associated with phenotype changes [353,364,365,366]. The “spontaneous” loss of knobs during long-term cultivation and the underlying genetic mechanisms have been studied for decades [367,368,369,370]. The underlying mechanisms are complex and still incompletely understood, and may be strain-dependent, including the selection of parasite population(s), point mutations, deletion, gene amplification, and differential transcription level. Adaptation, selection, and phenotypic and/or genotypic modifications may occur either simultaneously or in rapid succession and in a different order. There is still much work to be conducted for a better comprehension of malaria biology.

Given the large flexibility of the parasites at the individual level, the notion of clonality can be challenged. Right after cloning, when a single, isolated parasite in an infected erythrocyte multiplies “exponentially” after each intraerythrocytic cycle, some, but not all individual clones constituting the initially clonal population may undergo phenotypic and/or genotypic modifications, resulting in multiple populations, which are not genetically homogeneous. The individual clones may be subject to different cultivation conditions within the same culture vessel (e.g., in static cultures, those in the upper layer of the erythrocytes may have better access to nutrients and may have better gas exchange with the medium). Others may undergo mutations. Some of the resulting diverse populations may become more biologically fit and may be selected over time, leading to the emergence of a new population with different phenotypic and genotypic characteristics. Unless clonal populations are cloned regularly in a short interval of time, a clone may not remain a clone for a long time. One way to test this hypothesis is to compare the whole genome of the 3D7 clone used today in different laboratories around the world, which may or may not demonstrate its non-clonality.

Biological fitness is the ability of an organism to survive in its environment and reproduce and pass on its genes to the next generation of its offspring. The Darwinian concept of biological fitness and survival applied to the in vitro propagation of *P. falciparum* parasites may be juxtaposed with adaptation, selection, and phenotypic/genotypic modifications. Phenotypic features of a parasite strain related to higher parasite biomass, such as the multiplication rate, red blood cell selectivity (i.e., the number of multiply infected erythrocytes), the number of merozoites generated in each schizont, and merozoite invasion rate, are quantifiable variables that may reflect biological fitness of the asexual stage parasites [371,372]. In vivo, other phenotypic and genotypic features associated with antigenic variation and cytoadherence (the expression of *var*, *rifin*, and *stevor* multigene families; e.g., *P. falciparum* erythrocyte membrane protein 1, PfEMP1, encoded by the *var* gene family) play an important role in parasite survival and transmission [373,374,375]. These aforementioned parasite-associated factors that enhance biological fitness in the human host need to be counterbalanced because high virulence, defined as the ability to cause disease in the host, may lead to severe malaria and death, which is obviously unfavorable for parasite survival. A number of hypotheses have been advanced to explain these phenomena, but host–parasite interactions lie beyond the scope of this review and will not be discussed further. Suffice to say that there is evidence that biological fitness, the *P. falciparum* transmission rate, and virulence (i.e., malaria-associated morbidity and mortality) are positively correlated [376,377].

The biological fitness of drug-resistant *P. falciparum* isolates and strains has been studied extensively in the field and laboratory. The capacity to dominate or replace parasite strains during the in vitro co-cultivation of multiple parasite populations or strains has been studied, usually in the context of competition between drug-sensitive versus drug-resistant strains. Generally speaking, in the presence of selection (i.e., drug pressure), drug-resistant mutant parasites are likely to be fitter to propagate in a “hostile” environment, while in the absence of selection, the converse is more likely (i.e., drug-resistant mutant parasites are likely to be less fit than drug-sensitive wild type parasites) [378,379,380]. In vitro experiments have shown, however, that co-cultures initiated with equal numbers of chloroquine-sensitive and chloroquine-resistant clones in a drug-free medium lead to competition between the two clones and, after 11 days of continuous cultivation, the chloroquine-sensitive clone does not necessarily end up predominating the chloroquine-resistant clone [381]. When the same experiments were repeated with 50 nM chloroquine added to the culture medium (corresponding to > IC_50_ for the chloroquine-sensitive clone but much below the IC_50_ for the chloroquine-resistant clone), as expected, the chloroquine-resistant clone largely predominated over the chloroquine-sensitive clone, demonstrating the role of mutations associated with drug resistance in conferring biological fitness in an adverse environment.

In another experiment, it was shown that when compared to a drug-sensitive strain (HB3/Honduras), a multidrug-resistant strain (Dd2/Indochina) exhibited (i) a shorter intraerythrocytic cycle (44 h versus 50 h, respectively, in a standard culture medium supplemented with 0.5% Albumax^®^ II and cultivated in 5% CO_2_, 5% O_2_, and 90% N_2_), (ii) a higher number of merozoites per schizont (mean number, 18 versus 16 merozoites per schizont), and (iii) a higher invasion rate (i.e., a higher number of re-infected erythrocytes) underlying its faster growth rate [236]. It is not yet clear whether *P. falciparum* has a “biological clock” to regulate its metabolic activities on its own or whether the mammalian host imposes rhythmicity on the parasites, including the duration of the intraerythrocytic cycle [382,383]. A study has suggested that the mammalian host’s circadian rhythm may be controlling the synchronicity of the asexual intraerythrocytic life cycle of malaria parasites, possibly via melatonin or its precursors (e.g., serotonin) [384]. Another study has suggested that the rhythmic life cycle of each *P. falciparum* strain may be genetically programmed and driven by its endogenous “cell-cycle oscillator”, independent from the human host [385]. Experiments have shown that the intrinsic duration of the intraerythrocytic cycle may vary from 36 to 54 h, depending on the *P. falciparum* strain cultivated in vitro in standard conditions (suspension cultures using RPMI 1640 supplemented with 10% human serum, 5% O_2_, 5% CO_2_, and 90% N_2_ at 37 °C), but naturally occurring parasites adapt and align themselves with the 24 h host circadian biological clock. The precise molecular mechanisms are still under study, but similar findings were reported on *P. chabaudi* in mice [386]. The observation that the duration of the intraerythrocytic cycle of a parasite strain can be shortened or prolonged by changing the culture conditions (e.g., O_2_ concentration) may suggest that it is more of a survival strategy in response to an environmental change, rather than a “fixed” phenotypic/genotypic characteristic of a given parasite strain [214,215,266,267]. Of the three main differential phenotypic factors reported by Reilly et al. [236], the number of merozoites produced by individual schizonts and invasion rates seem to be the determinant factors directly related to differential growth rates, i.e., biological fitness, in different parasite strains. The underlying genetic basis of these phenotypic features of biological fitness is still under study [387].

Many factors that influence parasite growth in vitro were comprehensively reviewed in this paper. In some areas of research, more experimental data are required for a better comprehension of malaria biology. One such area is the interplay between adaptation, selection, phenotypic and genotypic modifications, rhythmicity (or chronobiology), and biological fitness. There are reasons to believe that laboratory-adapted *P. falciparum* strains or clones may not totally behave or respond like the parasites circulating in nature. If possible, experiments conducted in the laboratory using *P. falciparum* clones and strains need to be validated using field isolates. As a final word, with all its advantages and (some) disadvantages, the in vitro cultivation of *P. falciparum* is a model system that has been, and will continue to be, an essential tool for malaria research in the coming years.

## 16. Conclusions

The development of the technique for the continuous in vitro cultivation of asexual blood stages of *P. falciparum* is one of the major advances in malariology in the 20th century. The technique, developed in parallel and independently by Haynes et al. [26] and Trager and Jensen [27], has allowed several generations of scientists to work directly with the human pathogen and reduced or eliminated their dependence on (i) short-term, suboptimal cultures of *P. falciparum*, (ii) useful but less relevant avian, rodent, and simian malaria models, and (iii) experimentally infected monkeys as sources of parasites. It has provided a large amount of parasite materials for biological, biochemical, immunological, and pharmacological studies. Most fields in malaria research have been profoundly influenced and benefited from continuous cultivation techniques [13]. The basic principles laid out by Haynes et al. [26] and Trager and Jensen [27] remain valid and are largely unchanged more than 45 years after their discovery. Still, there are some areas where improvements are possible.

RPMI 1640 is the standard medium for *P. falciparum* cultivation. However, this medium was not specifically designed for malaria cultivation. Other media may support parasite growth as well as, or better, than RPMI 1640. Further studies with alternative media may be warranted. The requirement for human serum is a major disadvantage in the original technique described by Trager and Jensen, especially in developing countries. The use of Albumax^®^ as a substitute for human serum represents a major step toward the development of a serum-free, chemically defined medium. Further studies may identify the essential fatty acids required for optimal propagation of the parasites, foregoing even the use of Albumax^®^. At present, human erythrocytes are required for parasite propagation.

Lastly, the unfinished work of the late Professor William Trager on axenic culture should inspire and encourage young scientists to pursue this line of work. When some of these modifications, notably a serum-free culture system, are successfully attained, the cultivation of *P. falciparum* may become a routine procedure, even in developing countries where malaria is endemic.

## Figures and Tables

**Table 1:** Comparison of culture conditions for the long-term cultivation of *P. falciparum*.

Culture Conditions	Haynes et al. [26]	Trager and Jensen [27]
Culture medium	Medium 199 with Earle’s modified salts ^1^	Roswell Park Memorial Institute (RPMI) 1640
Buffer	10 mM TES ^2^	HEPES + NaHCO_3_ ^3^
Serum supplement	10% heat-inactivated fetal bovine serum	15% type AB human serum ^4^
Initial source of infected erythrocytes ^5^	Chimpanzee (*Pan troglodytes*)	Owl monkey (*Aotus trivirgatus*)
*P. falciparum* strain	Malayan Camp	FVO
Successfully tested erythrocytes	Chimpanzee, owl monkey, human	Human
Atmosphere	3% CO_2_, 6.6% O_2_	7% CO_2_, 1% or 5% O_2_; candle jar ^6^
Temperature	37 °C	38 °C
Culture vessel	5 cm^2^ flask, 96-well microtiter plate	Flow vial, Petri dish

^1^ Enriched with d-glucose (2 mg/mL), 2 mM l-glutamine, 3 × 10^−5^ M 2-mercaptoethanol, (±)-α-tocopherol (30 µg/mL) (note: the units of concentration are those of the authors being cited); ^2^ N-tris-(hydroxymethyl)methyl-2-aminoethanesulfonate (TES); ^3^ 25 mM each, N-(2-hydroxyethyl)piperazine-N′-(2-ethanesulfonic acid) (HEPES); ^4^ type AB was initially used to avoid agglutination when mixing human and simian (*Aotus trivirgatus*) erythrocytes; ^5^ before the development of long-term in vitro cultivation techniques, several *P. falciparum* isolates from patients (tourists or military personnel) were passaged and maintained in splenectomized monkeys (chimpanzees or owl monkeys) in the United States of America. Haynes et al. [26] initiated cultures from aliquots of cryopreserved infected chimpanzee erythrocytes. ^6^ The candle jar produces an atmosphere of approximately 3% CO_2_ and 17–18% O_2_.

**Table 2:** Sera from animal sources for the in vitro cultivation of *P. falciparum*.

Animal Sera ^1^	Parasite Growth ^2^	References
Fetal calf	good ^3^	[26,98,155]
	poor to fair	[81,101,156]
Calf	poor	[156]
	very good (with peptones)	[155]
	very good ^4^	[30]
	very good ^5^	[174]
	very good ^6^	[175]
Adult cow	poor to no growth (unsupplemented)	[93,156,169]
	fair (with neopeptone)	[93,156,169]
	very good (with hypoxanthine)	[176]
	very good ^6^	[175]
Rabbit	very good (may require adaptation)	[99,177,178] [179] ^7^
Goat	fair (for 96 h), poor > 96 h	[93]
	fair to good up to 30 days	[169]
	poor for three cycles	[99]
	comparable to 10% fetal bovine serum	[180] ^8^
Pig	poor (data not shown)	[29]
	poor (agglutination and hemolysis)	[156]
	very good for 96 h ^9^	[93,155]
	poor to fair (strain-dependent)	[169]
Horse	very good (without adaptation)	[29,99]
	poor	[156]
	poor for 96 h; no growth beyond 96 h	[93,155]
	fair to good up to 30 days	[169]
	very good after adaptation	[175]
Sheep	poor to fair (data not shown)	[29]
	poor	[156]
	poor to no growth	[93,155]
	poor during adaptation; fair to good after ^10^	[181]
	poor to fair (parasite strain-dependent)	[169]

^1^ Most experiments were performed with 10% (*v*/*v*) animal serum in the RPMI 1640 medium. ^2^ Relative terms are used in this table: “very good” if parasite growth is similar to human serum, “good” if parasite growth is approximately ≥75% compared with human serum, “fair” for parasite growth 25–75%, and “poor” for growth <25%. Because different factors (days of culture, different serum batches, parasite strains) used by the investigators may influence the results, readers are advised to consult the original references for details. ^3^ To obtain parasite growth similar to human serum, additional supplements (hypoxanthine, neopeptone) may be required [155]. ^4^ These investigators used the medium 199 and 15–20% calf serum. The culture medium was changed twice daily. ^5^ Supplemented with human erythrocyte extract or proteose peptone. ^6^ After adaptation with a glucose peptone supplement. ^7^ In this experiment, a higher amount of rabbit serum (15%) was added to RPMI 1640 [179]. ^8^ The author reported that two *P. falciparum* strains were adapted to the medium supplemented with 5–10% goat serum in long-term cultivation. ^9^ Long-term cultivation failed, partly due to agglutination. ^10^ Poor during the adaptation period of primary isolate (first 57 days of cultivation); fair to good thereafter (culture terminated on day 74 of this study) [181].

**Table 3:** Serum substitutes for *P. falciparum* cultivation.

Substitute	Composition	Comments	References
Bovine or human serum albumin	1 g/L fatty acid-free bovine or human serum albumin	Pre-Trager cultivation method; very good growth over 48 h	[185,186]
	Fatty acid-free bovine or human serum albumin (concentration not cited)	Unsatisfactory growth with the candle jar method	[156]
	5 g/L bovine serum albumin + 10% dialyzable human serum factors	No growth beyond 24 h	[187]
	6 g/L fatty acid-free bovine albumin + 10 mg/L adenosine	Poor growth (culture terminated on day 13)	[188]
	5 g/L fatty acid-free bovine albumin or 5 g/L Cohn fraction V of bovine albumin	Good for short-term (3–5 days) culture; better growth with Cohn fraction V	[54]
	0.4 or 4 g/L bovine serum albumin	Poor growth in the GIT medium ^4^	[101]
Lipid	Stearic acid	Pre-Trager cultivation method; fair growth at 48 h	[185,186]
	Stearic, palmitoleic, elaidic acids + bovine albumin	Poor growth with Trager’s method	[188]
	*Cis*-vaccenic, oleic, and linoleic acids (10 µM), individually or in combination + bovine albumin	Fair growth (about 50% of that observed with human plasma); successful long-term cultivation	[188]
	Lipid–cholesterol-rich mixture + Cohn fraction V of bovine albumin	Very good growth; suitable for long-term cultivation	[54]
Peptones	Bacto-peptone, Yeastolate, lactalbumin hydrolysate, bovine insulin, polyvinylpyrrolidone	no growth	[156]
	0.15% Neopeptone^®^ or Proteose-Peptone^®^ no. 3	Fair growth after adaptation; good growth when combined with fetal calf serum	[155]
	0.04% proteose peptone	Very good growth if supplemented with 10% calf serum	[174]
Human serum fractions	5 mg/mL bovine serum albumin + 10% dialyzable human serum factors + HDL or LDL	Good growth at 48 h with 0.5 mg/mL HDL or 1.8 mg/mL LDL; long-term culture not performed	[187]
	0.25–0.5 mg/mL HDL fraction	Successful short-term culture comparable to 10% human serum	[189]
GF21	Ammonium sulfate fraction of adult bovine serum, insulin, transferrin, ethanolamine, sodium selenite	Very good growth in the GIT medium ^4^; successful long-term culture	[101]
Nutridoma-SR^® 1^	Albumin, insulin, transferrin, cholesterol, organic and inorganic compounds	At 4% (*v*/*v*), growth comparable with serum-supplemented RPMI; may be strain-dependent	[190]
		Nutridoma alone at 4%, poor growth; 1% Nutridoma + 0.25% or 0.5% Albumax^®^, very good growth	[191]
Ultroser^®^ G ^2^	Binding proteins, fatty acids, phospholipids, adhesion factors, hormones, vitamins, growth factors	1–4% Ultroser, no growth; ≤0.5% Ultroser + ≤2% human serum, moderate growth for 4 days	[192]
		2–4% Ultroser, no growth; 0.5–1% low growth on days 5–9	[193]
Albumax I or II ^3^	Lipid-enriched bovine serum albumin	No growth unless combined with 1% Nutridoma	[191]
		Very good growth	[54,99,122,167,194,195,196,197,198,199]

The following relative terms were used to classify the extent of the suitability of serum substitutes: very good growth, comparable with medium supplemented with human plasma or serum; good growth, ≥75% of growth observed with medium supplemented with human plasma or serum; fair growth, 25–75% of growth observed with medium supplemented with human plasma or serum; poor growth, ≤25% of growth observed with medium supplemented with human plasma or serum. ^1^ Boehringer (Mannheim, Germany). ^2^ Invitrogen Gibco BRL. The exact composition of the Ultroser^®^ G serum substitute is unknown. Its partial composition was determined by Blixt et al. [200]. ^3^ Invitrogen Gibco BRL. ^4^ The GIT medium is a 1:1 mixture of Iscove’s and F-12 media supplemented with 10% GF21. HDL, high-density lipoprotein; LDL, low-density lipoprotein.

**Table 4:** A comparison of partial pressures and/or concentrations of oxygen and carbon dioxide in in vivo and in vitro conditions.

Site	Oxygen (mm Hg)	Carbon Dioxide (mm Hg)
Atmospheric dry air ^1^	159 (20.95%)	0.2–0.3 (0.03–0.04%)
Human body ^2^		
Pulmonary capillaries	100–104 (13.2–13.7%)	40 (5.3%)
Arterial blood ^3^	95 (12.5%)	40 (5.3%)
Venous blood	40 (5.3%)	45 (5.9%)
Tissue (intracellular)	5–40 (0.6–5.3%)	46 (6.1%)
Candle jar ^4^	17–18% (range, 14.5–17.8%)	3% (range, 1.1–3.3%)
CO_2_ incubator	19–20%	5%

^1^ Nitrogen, 594–601 mm Hg (78–79%). ^2^ Values cited in Guyton and Hall [263] and Boron and Boulpaep [264]. The values in alveolar air are the same as pulmonary capillaries. The values in venous blood are the same as interstitial fluid. The partial pressures of N_2_ and H_2_O in the human body at sea level are 569–573 mm Hg (74.9–75.4%) and 47 mm Hg (6.2%) at 37 °C, respectively. ^3^ Partial pressure of oxygen decreases to 95 mm Hg in the arterial blood due to the mixture of oxygenated blood that passes through the alveolar capillaries and unoxygenated blood returning from the deep lung tissues away from the gas exchange areas. ^4^ Approximate values and ranges measured by several authors [27,81,107,115,262]. The remaining gas content is mostly nitrogen; there is a small quantity of water vapor in the candle jar.

## Data Availability

Not applicable.

## Abbreviations

ACD, acid–citrate–dextrose; *ast*, gene encoding aspartate transaminase; *atg4*, gene encoding cysteine protease autophagy-related 4; ATP, adenosine triphosphate; BES, N,N′-bis(2-hydroxyethyl)-2-aminoethanesulfonic acid; BME, basal medium Eagle; *clag*, cytoadherence linked asexual gene; CMRL-1066, the medium developed at the Connaugh Medical Research Laboratories; COI, complexity of infection; CPD, citrate–phosphate–dextrose; CPD-A, modified citrate–phosphate–dextrose with 0.2 g/L adenine; DHFR, dihydrofolate reductase; DHPS, dihydropteroate synthase; DMEM, Dulbecco’s modified Eagle’s medium; DOC2, double C2-like domain-containing protein; *eba-140*, gene encoding erythrocyte-binding antigen-140; G6PD deficiency, glucose-6-phosphate dehydrogenase deficiency; HDL, high-density lipoprotein; HEPES, N-2-hydroxyethylpiperazine-N′-2-ethanesulfonic acid; IC_50_, 50% inhibitory concentration; IMDM, Iscove’s modified Dulbecco’s medium; LDL, low-density lipoprotein; M-199, medium 199; MCH, mean corpuscular hemoglobin; MCV, mean corpuscular volume; MEM, minimum essential medium; MOI, multiplicity of infection; MSA-1, merozoite surface antigen-1; MSA-2, merozoite surface antigen-2; NCTC 135, the medium formulated at the National Cancer Institute, Tissue Culture section (Bethesda, MD) for the L929 strain of mouse cells; PABA, *para*-aminobenzoic acid; PCR, polymerase chain reaction; PfEMP-1, *P. falciparum* erythrocyte membrane protein-1, expression product of *var* gene; PSAC, plasmodial surface anion channel; *rif*, gene encoding repetitive interspersed family; RIFIN, repetitive interspersed family; ROS, reactive oxygen species; rpm, rotations per minute; RPMI 1640, Roswell Park Memorial Institute cell culture medium 1640 (RPMI 1640); SAG, saline–adenine–glucose; SAGM, saline–adenine–glucose + mannitol; SNP, single nucleotide polymorphism; SRPK1, serine/threonine protein kinase 1; *stevor*, subtelomeric variable open reading frame gene family; *surf*, surface-associated interspersed gene family encoding SURFIN protein; TES, N-tris-(hydroxymethyl)methyl-2-aminoethanesulfonic acid; VLDL, very-low-density lipoprotein; WHO, World Health Organization; WRAIR, Walter Reed Army Institute of Research.
